# Changes in tree functional composition across topographic gradients and through time in a tropical montane forest

**DOI:** 10.1371/journal.pone.0263508

**Published:** 2022-04-20

**Authors:** Selene Báez, Belén Fadrique, Kenneth Feeley, Jürgen Homeier

**Affiliations:** 1 Departamento de Biología, Escuela Politécnica Nacional del Ecuador, Quito, Ecuador; 2 School of Geography, University of Leeds, Leeds, United Kingdom; 3 Department of Biology, University of Miami, Coral Gables, Florida, United States of America; 4 Department of Plant Ecology, University of Goettingen. Goettingen, Germany; Chinese Academy of Forestry, CHINA

## Abstract

Understanding variation in tree functional traits along topographic gradients and through time provides insights into the processes that will shape community composition and determine ecosystem functioning. In montane environments, complex topography is known to affect forest structure and composition, yet its role in determining trait composition, indices on community climatic tolerances, and responses to changing environmental conditions has not been fully explored. This study investigates how functional trait composition (characterized as community-weighted moments) and community climatic indices vary for the tree community as a whole and for its separate demographic components (i.e., dying, surviving, recruiting trees) over eight years in a topographically complex tropical Andean forest in southern Ecuador. We identified a strong influence of topography on functional composition and on species’ climatic optima, such that communities at lower topographic positions were dominated by acquisitive species adapted to both warmer and wetter conditions compared to communities at upper topographic positions which were dominated by conservative cold adapted species, possibly due to differences in soil conditions and hydrology. Forest functional and climatic composition remained stable through time; and we found limited evidence for trait-based responses to environmental change among demographic groups. Our findings confirm that fine-scale environmental conditions are a critical factor structuring plant communities in tropical forests, and suggest that slow environmental warming and community-based processes may promote short-term community functional stability. This study highlights the need to explore how diverse aspects of community trait composition vary in tropical montane forests, and to further investigate thresholds of forest response to environmental change.

## Introduction

Climate change is expected to drive major shifts in tropical plant communities [1–4]. Given the tight relationship between plant traits and environmental variation [5, 6], understanding trait-based responses to climate may provide valuable insights into the processes that will shape the composition and functioning of plant communities under changing environmental conditions [7, 8]. Tropical montane ecosystems are critical repositories of biodiversity [9, 10] and deliver many valuable ecosystem services [11]. Unfortunately, tropical montane systems are also believed to be especially sensitive to ongoing environmental alterations [12]. Changing geographic distributions is one commonly observed response of montane species to warming [13, 14]. Indeed, many Neotropical montane forests are already exhibiting increasing relative abundances of tree species that were formerly distributed at lower elevations, presumably because these lower-elevation species are better adapted to hotter temperatures (i.e., are more thermophilic) than their high-elevation counterparts, and thus are able to increase in abundance or shift their ranges upslope (via increased recruitment at the leading edge and/or increased mortality at the trailing one) as temperatures increase [15–18].

In tropical montane forests, the functional composition of trees varies along elevation gradients. In general, tree species characterized by more conservative strategies for resource acquisition and use (e.g., higher leaf toughness, higher wood density [WSG], lower specific leaf area [SLA], and lower leaf nutrient concentrations) are more abundant at higher elevations, and species with more acquisitive strategies (e.g., higher leaf nutrient concentrations, higher SLA, and lower WSG) prevail at lower elevations [5]. These patterns are typically attributed to the steady adiabatic decrease of temperature with elevation [5] along with decreasing nutrient availability [5, 19]. As such, anthropogenic global warming is expected to push the functional composition of tropical mountain plant communities towards greater relative abundances of species with more acquisitive traits [20]. In other words, changing species distributions due to warming have the potential to modify not only the floristic composition of communities, but also their functional composition (i.e., the relative abundances of acquisitive vs. conservative species) [20, 21], which in turn can affect community dynamics and ecosystem functioning.

Changing precipitation rates can also have profound effects on plant communities [22, 23]. Along tropical elevation gradients, the influence of precipitation on tree functional composition appears to be weaker than that of temperature [5]. This may be due in part to the fact that precipitation rates do not change consistently or predictably across elevations [24], as well as to the difficulty of translating precipitation to biologically meaningful metrics of plant water availability. That said, since climate change is affecting temperature and precipitation in these ecosystems [25, 26], both factors clearly need to be considered if we hope to understand the responses of tropical tree species and communities to climate change.

While the abundance of low-elevation, thermophilic tree species is increasing in most Andean forests [16–18], the rates of change are highly variable [18]. This suggests that some biotic and abiotic factors prevent range shifts in certain plant communities [18, 20, 27].

Topographic variation is a critical driver of structure, composition and dynamics in tropical forests [28–31]. At a given elevation, topography shapes plant communities through a variety of mechanisms, including through effects on hydrology, nutrient availability, temperature, wind exposure, and biological interactions [32]. Bottomland and lower slope areas usually have greater soil moisture and nutrient concentrations than the upper slopes and ridges [33–35]. In addition, valleys and lower topographic positions often experience cooler temperatures relative to upper positions due to cold air drainage and reduced sun exposure [36, 37], although the opposite patterns [38, 39] or no trends of temperature variation from lower to upper topographic positions have also been reported for some tropical montane forests [35]. These differences in resource availability and micro-environmental conditions can have major effects on taxonomic and functional trait composition such that tree communities in lower topographic positions typically include more species with acquisitive traits, whereas upper positions and ridges are usually dominated by species with conservative traits [29, 34, 40]. However, it remains unknown how these patterns of plant trait distribution driven by local topographic variation relate to the species climatic preferences considering regional gradients of elevation.

At a particular site, topographic variation may modulate forest responses to climate change through various mechanisms. Topography may strongly regulate small-scale climate variation and plant community composition in montane environments [38, 41, 42], prevent some species from tracking climates [18, 27], and define the location of climatic refugia for some species [43–45]. Under increasing temperatures, the tree communities in lower topographic positions that harbor acquisitive species with a greater potential for fast resource uptake and investment [46, 47] may be expected to respond more-strongly in terms of growth than communities at upper positions that have more conservative species. In contrast, higher temperatures could promote the colonization of more acquisitive species into habitats at upper positions, and thereby affect the floristic composition of these communities. Thus, trait-based responses of different demographic groups can indicate the mechanisms that determine plant community responses to climate change along topographic gradients. Particularly marked differences in trait composition may occur between mortality and recruitment demographic groups, as dying trees were established under environmental conditions likely different than present ones, under which new recruits are succeeding.

We investigated how functional trait composition and community climatic indices vary across spatial and temporal gradients in a topographically complex tropical montane forest in the Andes of Ecuador. Under the premises put forward by Trait Driver Theory that sustained trait-based responses to directional environmental variation can be described by community-weighted means (CWM) [48], we focused our analyses on exploring how CWM vary in response to environmental gradients, through time and across tree demographic groups. Following the same theoretical context, to have a broader understanding of trait distribution within the studied tree communities, we explored how three additional community-weighted moments: variance (CWV), kurtosis (CWK) and skewness (CWS) varied across topographic gradients and time [48]. Using data collected over an eight-year period, we tested the hypotheses that 1) topography affects tree community trait composition from lower to upper topographic positions, which may reflect on community climatic indices and the relative abundance of tree species with acquisitive vs. conservative traits, and 2) community thermal and precipitation preferences may be increasing through time, such that communities will exhibit increasing relative abundance of species with acquisitive strategies and with higher thermal optima, due to the upslope shifts in the ranges of species. Understanding the role of topography on tree functional trait distribution and climatic optima is critically needed to help explain the observed variation of tropical montane forest sensitivity to climate change.

## Results

In total, we measured 1594 dicotyledonous trees in our 18 plots—1341 individuals in the first census, and 1315 individuals in the last census, 1062 trees stayed alive over the eight-year census period, 279 individuals died, and 253 individuals were recruited. The average annual turnover rate for individuals was 2.87 (± 0.29) %. Of the measured trees, 1425 (89.4%) were identified to species, 105 (6.6%) were identified to genus, 49 (3.0%) were identified to family level. Fifteen individuals (1.0%) remained undetermined. In total, we identified 220 species or morphospecies.

### Floristic composition, topography and time

**Fig 1:**
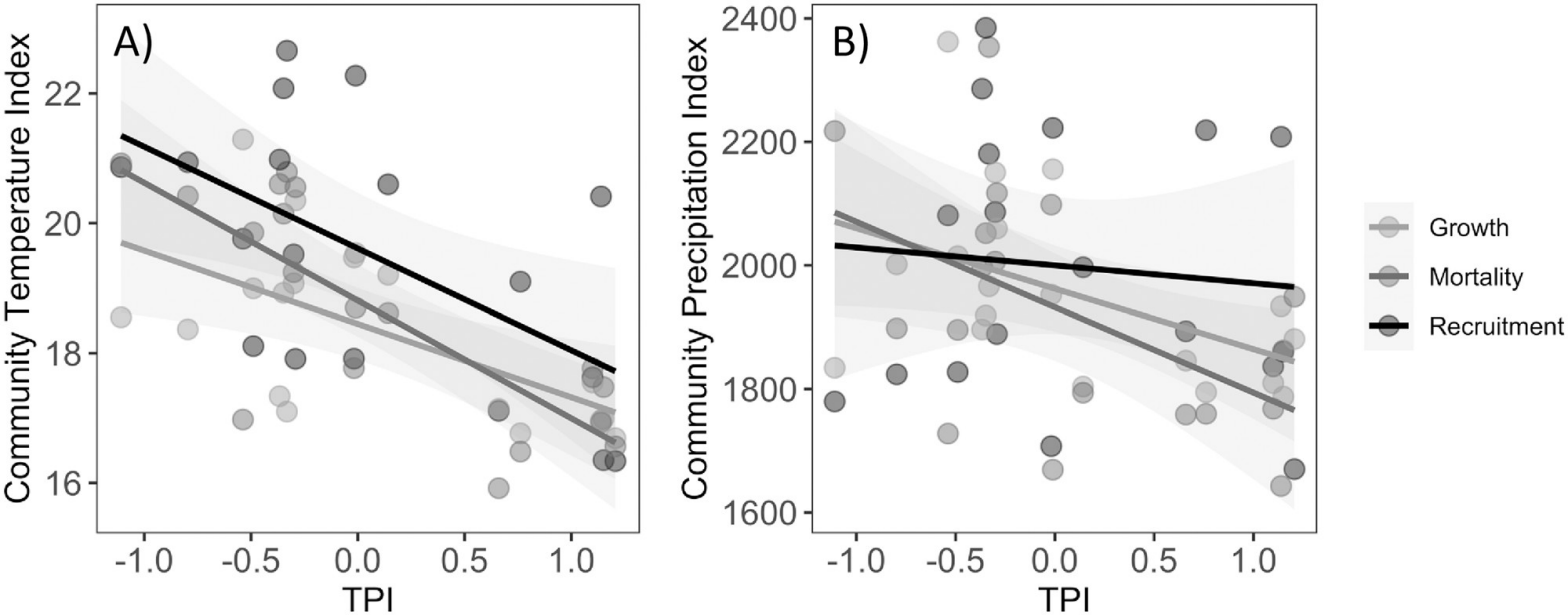
Non-metric dimensional scaling (NMDS) of tree species composition along a topographic gradient measured as Topographic Position Index (TPI). A) Indicates variation in species composition during the first monitoring, B) indicates differences in floristic composition between dead and recruited summarized in a NMDS axis along the topographic gradient. Each point represents a forest monitoring plot.

The floristic composition of the plot communities during the first monitoring year was strongly affected by Topographic Position Index (TPI) (PERMANOVA, *F*_1,17_ = 4.54, R^2^ = 0.22, *P* = 0.001, Fig 1A, S1 Fig). Floristic composition did not change over time (time: *F*_1,35_ = 0.06, R^2^ = 0.002, *P* = 1.00) nor through time across the topographic gradient (TPI x year: *F*_1,35_ = 0.07, R^2^ = 0.002, *P* = 1.00). However, the recruited and dead trees did differ significantly in their species composition (*F*_1,35_ = 1.68, R^2^ = 0.04, *P* = 0.004), and this shift was dependent on the topographic position of the plot (TPI x demographic group: *F*_1,35_ = 1.37, *R*^*2*^ = 0.04, *P* = 0.028). Specifically, plots at the lower and upper positions had greater rates of change in their floristic compositions over time than did communities at mid topographic position (Fig 1B).

### Trait composition, community climate indices, and topography

**Fig 2:**
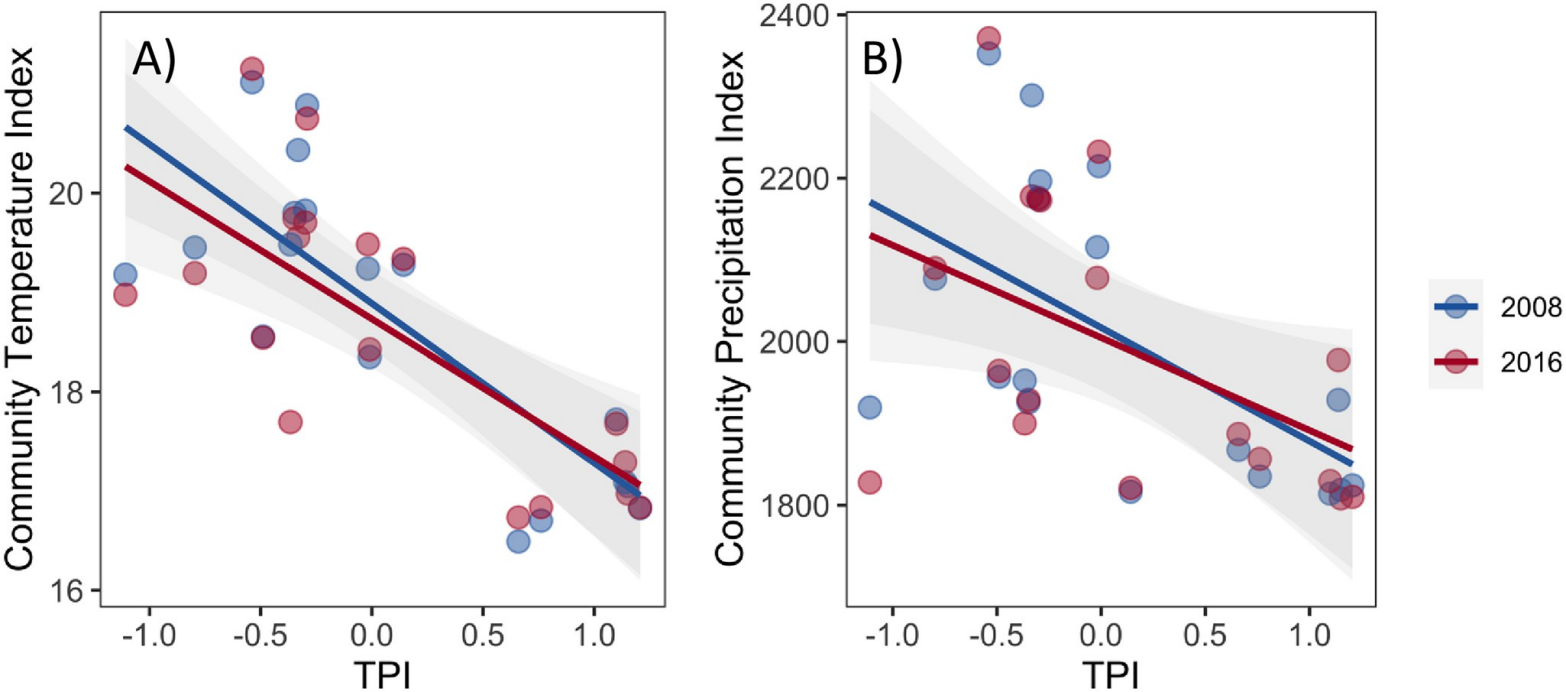
Scaled Community Weighted Means (CWMs) of leaf and stem functional traits as a function of topographic variation Topographic Position Index (TPI). **A**ll traits except for bark thickness and sapwood-specific conductivity varied significantly with TPI (S2 Table). The slopes of the linear regression lines represent the first (2008) and last (2016) sampling years were not significantly different for any trait. Each point represents a forest monitoring plot.

**Fig 3:**
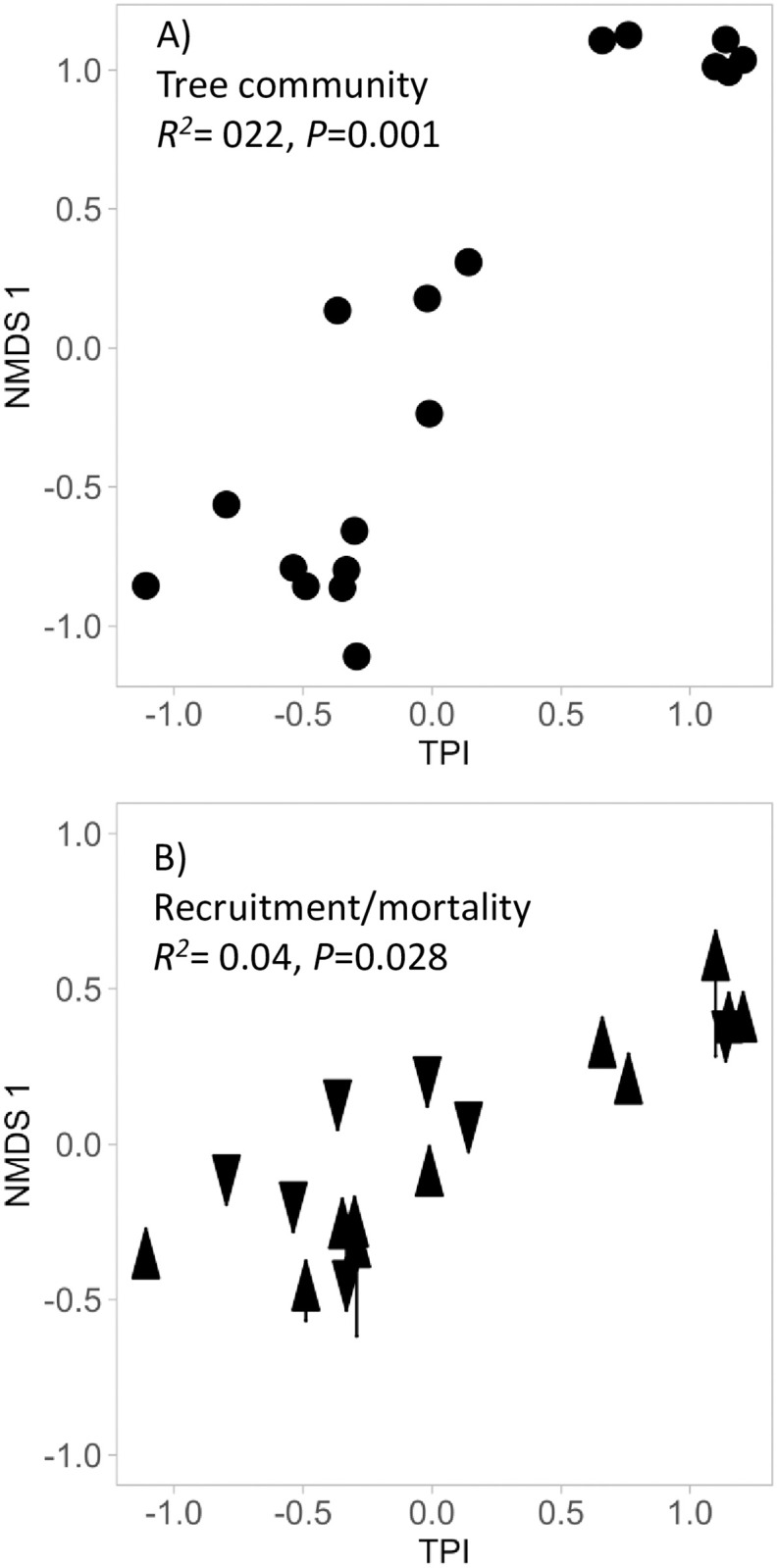
Summary of variation in community functional trait composition and climatic indices along a topographic gradient.

Topography significantly affected the community trait composition (CWMs) of eight functional traits (Table 1, Figs 2 and 3, S1 and S2 Tables). At lower TPI values, the tree communities had high average bark thickness, leaf N and P content, leaf area (LA), specific leaf area (SLA), sapwood-specific conductivity (KS), vessel diameter, and low average values of leaf toughness, vessel density and wood density (WSG). At higher TPI values (towards upper positions), this pattern of trait composition reversed, reflecting the expected gradient of acquisitive to conservative strategies of plants along the topographic gradient.

**Table 1:** Functional traits used in this study, their relation to plant performance after van der Sande et al 2016 [47], and trends of variation along topographic gradients reported in the literature or in this study for tropical forests.

Description / abbreviation	Units	Related to	Values in relation to topography from lower to upper slopes	Reference
Bark thickness [BT]	mm	stem defense	high—low	This study
Leaf area [LA]	cm^2^	light interception, heat balance	high—low	Kraft et al. 2010
Leaf toughness [LT]	kN m^-1^	leaf defense	low—high	This study
Foliar nitrogen [N]	mg g^-1^	photosynthetic capacity	high—low	Werner and Homeier 2015, Kraft et al. 2010
Foliar phosphorus [P]	mg g^-1^	growth and photosynthetic capacity	high—low	Werner and Homeier 2015, Kraft et al. 2010
Sapwood-specific conductivity [KS]	kg m^-1^ Mpa^-1^ s^-1^	conductivity of fluids, drought tolerance	high—low	This study
Specific leaf area [SLA]	cm^2^/g	light interception efficiency	high—low	Kraft et al. 2010
Vessel density [VDen]	units/mm^-2^	conductivity of fluids, drought tolerance	low—high	This study
Vessel diameter[VDia]	μm	conductivity of fluids, drought tolerance	high—low	This study
Wood density [WSG]	g/cm^3^	Stem defense, drought tolerance	low—high	Valencia et al. 2009, Kraft et al. 2010

In our data set, the functional traits and climatic optima of species were correlated. Leaf N and P concentrations, SLA, and vessel diameter correlated positively with species’ optimal mean annual temperature, whereas leaf toughness and vessel density correlated negatively with species’ optimal mean annual temperature (Table 2, see S3 Table for a full trait and community climatic indices correlation matrix). On the other hand, SLA and vessel density correlated in a positive and negative fashion, respectively, with species’ mean total annual precipitation (Table 2).

**Table 2:** Non-parametric correlations between species functional traits, and species climatic optima, and number of species included in the respective analyses. Statistically significant values are presented in bold.

Functional trait	Species thermal optima		Species precipitation optima		Species
	Spearman p	*P*	Spearman p	*P*	n
Bark thickness [BT]	-0.06	0.538	-0.16	0.081	106
Leaf area [LA]	0.22	**0.033**	0.02	0.824	89
Leaf toughness [LT]	-0.22	**0.033**	-0.08	0.454	89
Foliar nitrogen [N]	0.31	**<0.001**	0.16	0.093	110
Foliar phosphorus [P]	0.32	**<0.001**	0.14	0.118	110
Sapwood-specific conductivity [KS]	0.14	0.134	0.10	0.242	115
Specific leaf area [SLA]	0.37	**<0.001**	0.27	**0.006**	98
Vessel density [VDen]	-0.22	**0.014**	-0.19	**0.038**	115
Vessel diameter[VDia]	0.18	**0.046**	0.13	0.136	115
Wood density [WSG]	0.03	0.738	0.11	0.208	118

The repeated linear mixed effect models indicated that CWVs, CWKs and CWSs were less affected by topographic variation than CWMs (S4 Table and S2 Fig). Community-weighted variance (CWS) of leaf P concentration, SLA, and KS and vessel diameter decreased toward higher topographic positions, whereas variance of leaf toughness had the opposite pattern. Community-weighted skewness (CWS) decreased for leaf P concentration toward areas with higher TPI values. Community-weighted kurtosis (CWK) was not significantly affected by topography.

**Fig 4:**
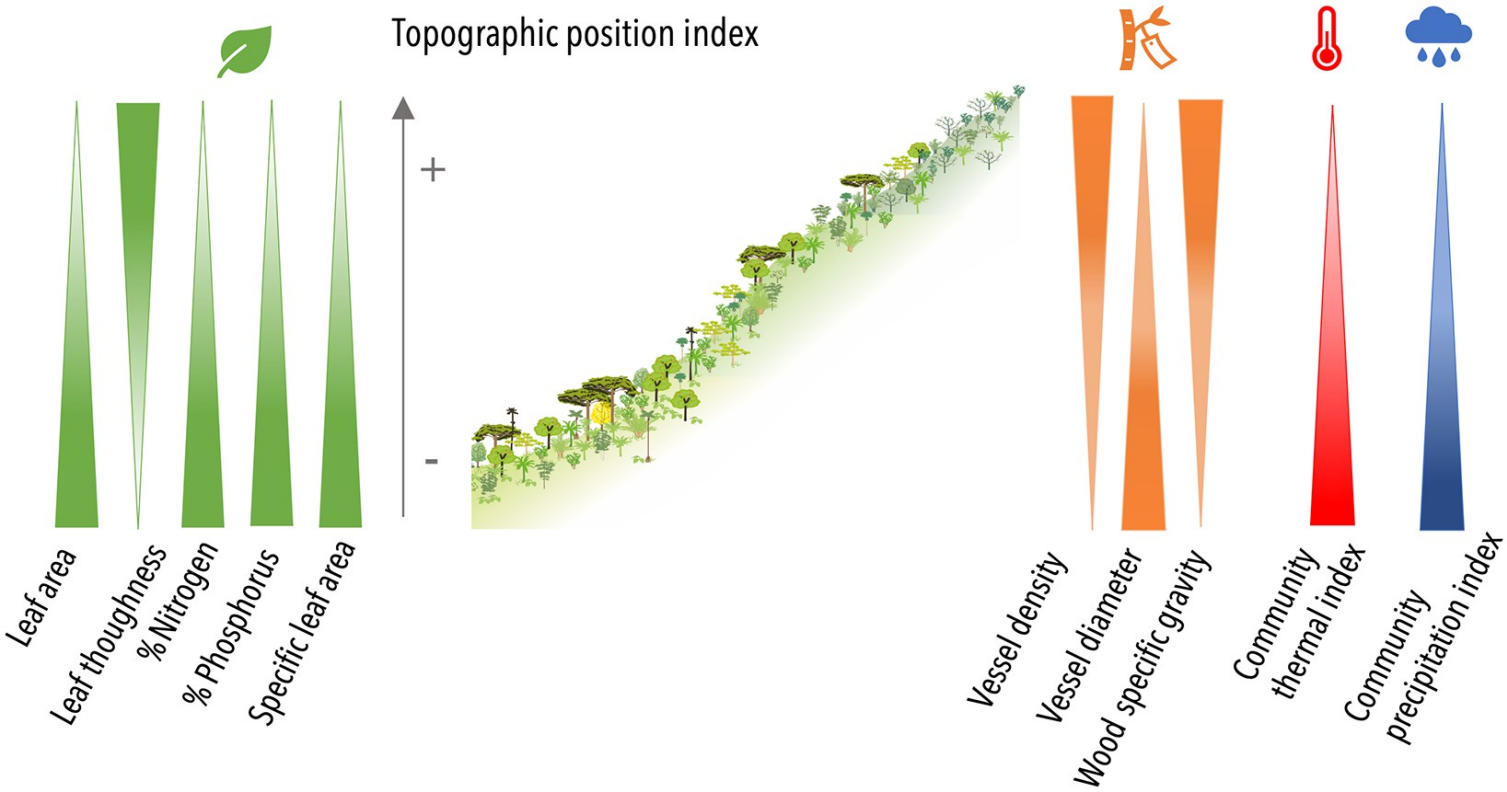
Community Temperature Index (A) and Community Precipitation Index (B) for the plant community along a topographic gradient Topographic Position Index (TPI). Lines indicate statistically significant linear regressions (*P*≤0.05) for the first (2008) and last (2016) monitoring year. Each point represents a forest monitoring plot.

For our study plots Community Thermal Index (CTI) significantly decreased with TPI. This indicated that plots at lower topographic positions had higher relative abundance of species with hotter thermal optima compared to the upper slope forests (Linear regressions: CTI yr1 = 18.91–1.52 x TPI, R^2^ = 0.57, P< 0.001, CTI yr8 = 18.67–1.36 x TPI, R^2^ = 0.54, P <0.001; Figs 3 and 4A, S2 Table). In fact, plots located towards lower topographic positions had CTI’s up to 4°C higher than those at upper topographic positions. Similarly, Community Precipitation Index (CPI) decreased with TPI, such that plots at lower topographic positions had greater relative abundances of species adapted to wetter environments (higher CPI) compared to plots located at upper topographic positions (Linear regressions: CPI yr1 = 1987.65–120.46 x TPI, R^2^ = 0.28, *P* = 0.024; CPI yr8 = 1967.07–107.25 x TPI, R^2^ = 0.23, *P* = 0.043, Figs 3 and 4B, S2 Table). Plots at lower topographic positions had CPI values that were up to 400 mm greater than those of communities located at upper topographic positions. Community-weighted skewness (CWS) increased along topographic position for CTI and CPI (S4 Table and S2 Fig).

### Community functional trait composition and climate indices through time

The CWMs did not change over the study period along topographic gradients for any of the ten functional traits that we measured (Fig 2, S2 Table). Similarly, CWMs of the demographic components of the tree community (i.e., dying, surviving and recruiting trees) had statistically significant different mean values in only one functional trait: vessel diameter (S5 Table and S3 Fig). Specifically, CWMs of stem vessel diameter of trees that stayed alive through the study period was significantly higher than of the community of recruits (Tukey HSD P<0.05).

**Fig 5:**
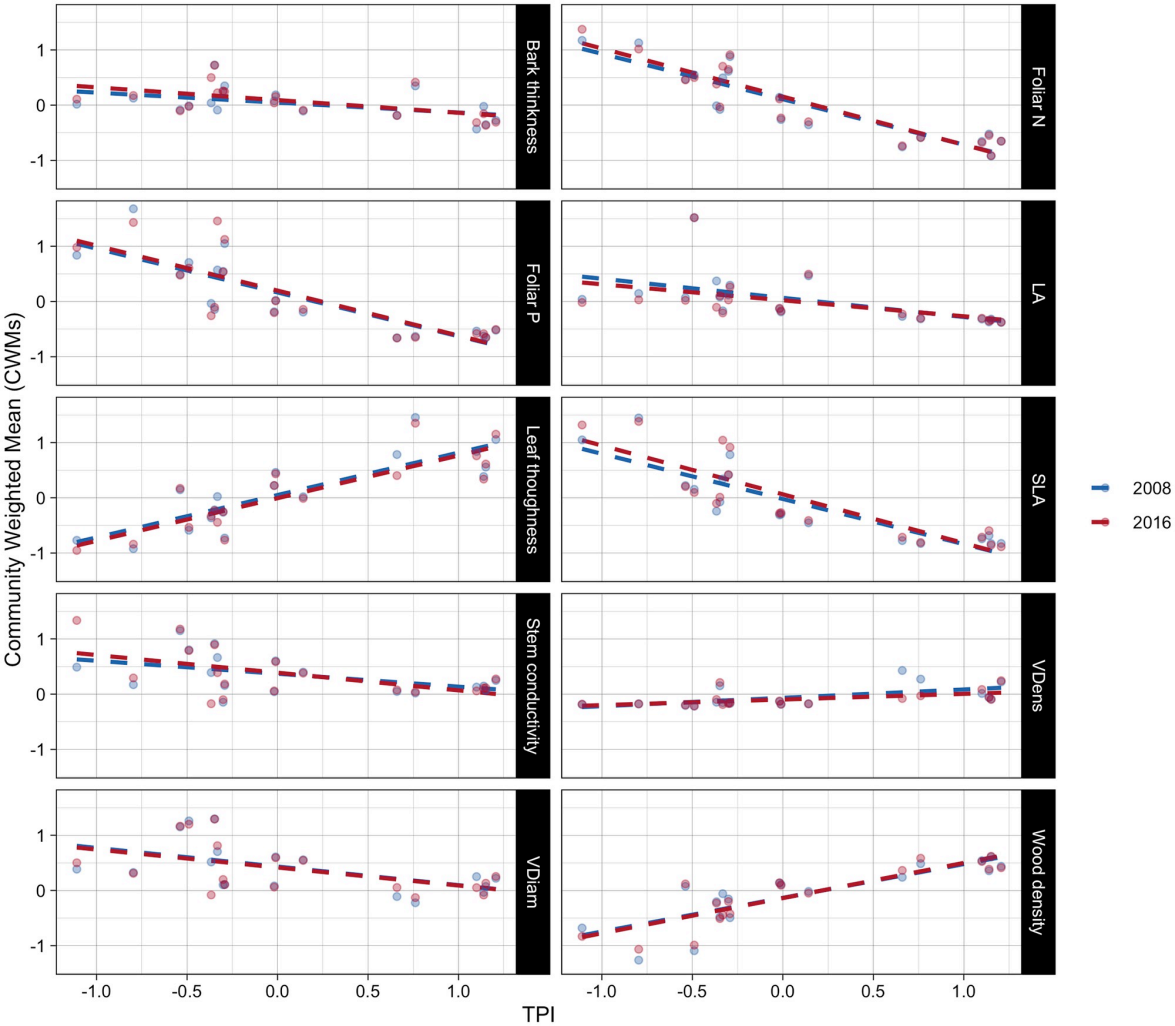
Community Temperature Index (A) and Community Precipitation Index (B) for the community’s demographic groups along a topographic gradient (Topographic Position Index = TPI). Lines indicate statistically significant linear regressions (*P*≤0.05). Each point represents a forest monitoring plot.

Our study plots did not experience directional changes of their Community Temperature Index (CTI) over the eight-year study period (TRplot mean = -0.02 ± 0.01 per year, binomial probability distribution *P* = 0.759). The mean CTI of the entire plant community did not change over time (Fig 4A, S2 Table), nor across the three demographic groups (Fig 5A), as they were only affected by TPI following the same trend of the entire plant community (S5 Table).

As with CTI, the Community Precipitation Index (CPI) of our plots did not experience directional shifts over time (PRplot mean = -1.26 ± 1.26 per year, binomial probability distribution *P* = 0.240), and their mean CPI did not change over time (Fig 4B, S2 Table). Demographic groups did not differ in their CPI either but responded to TPI as observed for the entire plant community (Fig 5B, S5 Table and S3 Fig). None of the three community-weighted moments (CWK, CWS, CWV) changed significantly over time in our forest monitoring plots (S4 Table and S2 Fig).

## Discussion

Our results indicated that the topographic position of our plots strongly influenced the floristic and functional composition of the tree community and revealed unrecognized links between topography and species climatic optima in tropical montane forests. Furthermore, our censuses and demographic analyses indicated that forest functional and climatic composition remained relatively stable at our study site over the eight-year study period. As discussed below, we propose that variation in soil conditions rather than temperature mediated the observed patterns of community trait composition and that functional stability may be driven by various factors, including high species diversity, the location of our study site at an ecotone, low rates of climatic warming, and topographic complexity.

In agreement with our first hypothesis, the observed gradient in CWMs of acquisitive to conservative traits in plots at lower- to upper- topographic positions was in line with previous research in tropical lowland and montane forests (Figs 2 and 3, S2 Table) [28–31, 40, 49] and supports the pattern of fine root traits found in the same study plots [50]. The lower values of CWVs toward high topographic positions for four of the examined traits were also in line with the trends observed for CWMs, and confirm the role of topography as an environmental filter [48]. It is interesting to note that functional trait composition measured as CWMs along topographic gradients (from lower- to upper positions) mirrored the patterns observed along large elevation gradients in tropical forests [5], emphasizing the role that local factors, and not just large-scale climate gradients, can play in structuring trait distribution in these ecosystems.

An unanticipated finding of this study was the strong relation between topographic variation and community climatic indices. Indeed, CTIs in plots at lower topographic positions were up to 4**°**C higher than in plots at upper topographic positions, which would correspond to a difference of ~700 m in elevation assuming a lapse rate of 5.5**°**C per 1000 m (in contrast to the 1**°**C range of CTI expected across our study plots which are separated by a maximum of just 176 m, Figs 3 and 4A). The higher CTI of forests in lower topographic positions contrasts with the idea that lower topographic positions and valleys experience decreased temperatures due to lower solar energy inputs, cold-air drainage and high canopy cover [36, 37]. For tropical humid montane forests, the only study we know of that reports field-recorded temperatures along topographic gradients did not find statistically significant differences between lower and upper topographic positions [35], suggesting that other factors (e.g., lower variation in aspect, wind) may homogenize environmental temperatures in these ecosystems. In consequence, although topography may have affected microclimatic conditions at our site, we think it is unlikely that these climate differences could have caused the gradients of up to 4**°**C in CTI and 400 mm in CPI, especially given the small differences in elevations between sites (Figs 3 and 4B). Rather, we hypothesize that topography is a filter for plant functional traits that correlate with the species’ thermal and precipitation optima. In particular, the effects of topography on local soil conditions and hydrology may influence the establishment of plant communities with varying requirements of water availability [22], which in turn relate to species’ thermal preferences. Indeed, seven of the ten functional traits analyzed had statistically significant correlations with species optimal temperature, and two with optimal precipitation (Tables 1 and 2).

The effects of topography on climatic composition may have been particularly strong at our study site, which was located at the upper elevational limit of lower montane forests [51]. In this transition zone, topographic complexity could facilitate the establishment of lower montane species at their maximum elevations, especially in lower topographic positions that are relatively sheltered and that have higher soil moisture and nutrient content. In contrast, upper topographic positions that are more exposed with poorer and periodically drier soils may be inhospitable for the lower-elevation species and favor stress-tolerant species from higher elevations that are less thermophilic and have lower precipitation optima. In fact, this process contributes to understand the positive effect of TPI on CWSs in the two community climatic indices (S4 Table and S2 Fig). For both of these (CTI and CPI), skewness became more positive (i.e., higher densities of low CTI and CPI values, left leaning distribution) toward high topographic positions, indicating that as TPI increased, the representation of species with higher thermal and precipitation optima decreased. Thereby, these results highlight the possibility that in montane forests topography may be a particularly powerful factor for structuring plant communities around ecotones, magnifying stress gradients and creating mosaics of species with different thermal and precipitation optima at fixed elevations, thereby enhancing community complexity at landscape scales.

Stable trait composition in spite of the observed floristic change (Fig 1B) probably occurred because the recruiting species were functionally equivalent to the species that died, suggesting that environmental pressures were not acting selectively for or against species with certain functional traits. Rather, these results suggest that we witnessed the dynamics of a diverse mature forest where tree species turnover did not cause directional changes of functional composition, possibly due to high species diversity and functional redundancy [52–54]. Functional redundancy is expected to enhance the resilience of ecosystems under changing environmental conditions [55], and may be buffering directional changes of primary productivity in Neotropical montane forests where CTI is increasing due to rising temperatures [56]. The fact that our individuals turnover rates fall within the range of those estimated for humid tropical Andean forests [57] also suggests unaltered natural forest dynamics.

High rates of species turnover at upper and lower TPI sites were most likely not a result of higher environmental temperatures as we did not detect increasing CTIs (Fig 4A) or higher representation of species with acquisitive traits (Fig 2). Rather, the rapidly increasing rates of nutrient deposition in our study area [58, 59] could have disadvantaged species adapted to poor soils and to a lesser extent facilitated the colonization of species adapted to more fertile soils [60]. At lower topographic positions, higher rates of floristic change may be a result of higher rates of turnover of individual trees compared to upper positions [29, 30, 58].

It is noteworthy, that the only trait difference between demographic groups that we found was the larger vessel diameter in surviving trees vs. recruited, which suggests that recruiting individuals are of species that sacrificed rapid resource uptake for protection from water deficits that may cause embolism [61]. Our study area experiences short dry spells every few years, which may affect tree recruitment; but it is unknown whether the frequency and intensity of these events is shifting due to climate change. More appropriate comparisons across demographic groups will require longer monitoring periods in which the recruiting trees have experienced a fuller range of biotic and abiotic constraints that ultimately determine the species composition of the entire plant community, or comparisons of the composition of the community demographic groups between forests censuses spanning shorter periods.

Finally, rates of climate warming in Andean forests ecosystems are predicted to be highly variable across elevations [26], potentially contributing to the heterogeneous rates of migration and functional change observed in forests throughout the Andes [18]. In this context, our study site seems to have experienced comparatively low levels of climate change and environmental forcing during our study period, perhaps below a response threshold, resulting in stable community climate indexes and functional trait composition especially given that our study was conducted over just an eight-year monitoring period. Indeed, the estimated annual temperature increase of 0.013ºC for our study area [62, 63] lies well below the Andes-wide average warming rate of 0.6ºC [18]. Finally, complex topography has been demonstrated to contribute to lower rates of warming in montane areas [37, 64]. Therefore, a combination of biotic and abiotic factors may contribute to functional stability in our study sites.

Micro-environmental conditions are increasingly recognized as critical drivers of plant responses to climate change [44, 65–67]. Indeed, in montane environments, fine-scale environmental heterogeneity may mediate the reorganization of plant communities under climate change in ways that do not necessitate shifts in species’ altitudinal distributions, but rather follow specific combinations of topographic and micro-environmental conditions [41]. This notion emphasizes the need of intensive and long-term vegetation and micro-climate monitoring at landscape-scales to explore multidimensional forest responses that depart from upslope and poleward expectations of species movements under climate change [45, 68], especially in topographically complex and species-rich tropical montane forests.

While our eight-year monitoring period is a relatively small window of time over which to document forest responses to climate change, previous research has found changes in the community climatic indices in Andean forests over periods as short as four years [17]. Overall, slow environmental warming, community-based processes, fine-scale environmental conditions, and the location of our plots within a transition between forest types all appear to have resulted in community functional stability. In a broader sense, our findings highlight the need to investigate forest response thresholds and how factors beyond climate affect tree trait distributions in tropical montane forests and present a cautionary call for studies exploring trait composition along elevation gradients as they may suffer from systematic topographic sampling biases at different altitudes.

## Methods

### Study site

This study was conducted in montane forests on the eastern slopes of the Andes of southern Ecuador (Reserva San Francisco, 3**°**58’S, 79**°**04’W) in a transition area between lower and upper evergreen montane forests [51]. The area has a mean annual temperature (MAT) of 15**°**C and receives a mean total annual precipitation (MAP) of 2100–2200 mm. The area has been warming at the rate of 0.13ºC per decade since the early 1960s, but there have been no directional changes of precipitation [62, 63]. The atmospheric deposition of nitrogen and phosphorus has increased during the last decades [58, 59].

The study area has a complex topography with slopes of varying degrees of steepness. Soils are heterogeneous but are generally poor in nutrients [29, 30, 69, 70]. Here, as in other tropical forests, topography strongly affects soil nutrient concentrations and hydrology, with soils at lower topographic positions exhibiting substantially higher concentrations of nitrogen, phosphorus and micronutrients, which affects species composition, tree trait composition, forest turnover and productivity rates [29, 30, 69, 71].

### Experimental design and field measurements

In 2008, eighteen permanent plots measuring 20 x 20 m each were established in a narrow altitudinal belt of < 180 m of elevation, between 1900 and 2100 m a.s.l. In the plots, we marked, identified, and recorded the diameter of all dicotyledonous trees ≥5 cm dbh (diameter at breast height). In 2016, we re-measured the trees in the plots, documenting tree death and recruitment.

The plots were distributed with a minimum separation distance of 50 m to capture topographic variation. For each plot we obtained a Topographic Position Index (TPI) derived from the relative position of the plot’s corresponding raster cell in a digital elevation model (DEM) related to the average elevation of the surrounding cells [72]. We used a DEM with 10 m resolution generated from stereo aerial photos by aerotriangulation [73] to calculate the TPI for a circular neighborhood around each plot with a radius of 200 m [74]. Negative TPI values indicated lower topographic positions, positive values, upper positions, and values around zero characterized middle-slope positions (S4 Fig). Our plots were distributed across a narrow range of elevation (mean elevation = 2003 m ± 50.84, min = 1913, max = 2089), but captured large variations in TPI (mean TPI = 0.08 ± 0.73, min = -1.11, max = 1.21).

Previous soil sampling in our study plots indicates that soil properties are highly associated with TPI [29, 30, 69, 71] (S5 Fig). Soil nutrient concentrations, including N, K, Mg, Ca, and plant-available P are higher at lower topographic positions and decrease toward upper topographic positions. In contrast, the depth of the organic layer and the soil C:N ratio increases toward upper positions.

### Tree functional traits

For 158 of the 200 species present in our monitoring plots, we measured ten leaf and stem traits expected to respond to topographic variation as indicated in Table 1. Trait measurements were taken between 2008 and 2014 from trees distributed in our study area, using standard methods as described in S6 Table. For individuals identified only to their family or genus, we used mean trait values of their lower taxonomic levels.

### Species climatic optima

We estimated the thermal and precipitation optima of the tree species present in our study plots based on the locations of observation and collection records relative to large-scale climate patterns following protocols modified from Fadrique et al. (2018) [18]. Specifically, we downloaded all available georeferenced records of the target species from the Andean countries of Venezuela, Colombia, Ecuador, Peru, Bolivia and Argentina from the Botanical Information and Ecological Network (BIEN) database [75]. We added distribution data from the BioWeb database [76] for those species that were absent or that had <20 records in BIEN. To minimize possible bias, we eliminated records with obvious georeferencing errors or that fell outside the Andean region and we only used one record per coordinate for each species to avoid including duplicates. Next, we extracted the mean annual temperature (MAT) and mean total annual precipitation (MAP) values at the record coordinates from the CHELSA extrapolated climate map [77] with a spatial resolution of 30 arcsec (approximately 1 km^2^ at the equator). We then calculated each species’ thermal optimum as the average of the extracted MAT values, and each species’ precipitation optimum as the average of the extracted MAP values. We obtained the thermal and precipitation optima for 154 (70% of the species pool) species present in our plots, representing on average 70% of the plots basal area (S7 Table), using a mean of 187 observations per species.

### Functional trait composition and community climatic indices

We used standardized trait values (mean = 0, standard deviation = 1) to calculate plot level community-weighted moments, a measure of community trait composition in which species’ trait values were weighted by their basal area in a plot (i.e., basal area = summed cross-sectional stem area of all conspecifics in a plot). We calculated community-weighted moments for the first and last year of our monitoring period to evaluate directional changes in overall community trait composition following the equations A16, A17 and A18 described in Enquist et al. 2015 [48]. In this way, we captured variation in community-level trait composition (i.e., CWMs), but also on community-level trait distribution (i.e., CWVs, CWSs, CWKs).

Community-weighted means (CWMs) were also calculated for three demographic groups: trees that died between censuses, trees that survived, and trees that were recruited during the census period to investigate the associations between these demographic processes and functional trait composition [78].

As we did with species functional traits, we calculated the community-weighted moments of the species’ thermal and precipitation optima in each plot (with species weighted by their plot basal area) to generate a community temperature index (CTI) and a community precipitation index (CPI) for each plot. We calculated the CTI and CPI for each plot for the first and last years of the monitoring period, and for the separate demographic subsets (i.e., dead, recruiting, surviving trees). For comparison, we also calculated CWMs using the number of stems per plot to measure trait representation in the community. Our results indicated that both ways of calculating CWMs yield highly correlated values (correlation coefficients between 0.73 and 0.99, and were highly statistically significant in most cases (S8 Table). We therefore conducted the statistical analyses using CWMs calculated with plot-scale basal area, because it better represents the contribution of the single species to aboveground biomass and ecosystem functioning.

### Data analyses

In our dataset, elevation and TPI were significantly correlated (pairwise correlation = 0.72, *P* = 0.001). Thus, we performed various statistical analyses using PERMANOVAs and linear mixed models that included elevation and compared them using R^2^ values and Akaike Information Criterion (AIC) [79] to evaluate the relative effect of elevation and topographic variation in our short topographic gradient. The approach and results of these analyses are presented in S9 and S10 Tables. Since these analyses consistently indicated that topographic variation was the main determinant of floristic and trait composition in our permanent plots, we performed subsequent statistical analyses excluding the elevation variable. We also tested for spatial autocorrelation in our trait measures to assess the need to include terms describing spatial distribution of the study plots in the statistical analyses [80]. There was no evidence suggesting spatial autocorrelation in our plot-scale trait measurements (see S11 Table for methods and results); we therefore developed further analyses without spatial correlation terms.

#### Floristic composition

To understand the effects of topography on species composition, we ran PERMANOVAs on Bray-Curtis species dissimilarity matrices using species plot scale basal area as a measure of species abundance, with 999 permutations in the ‘vegan’ statistical package [81] in R [82], and we used non-metric multidimensional scaling (NMDS) to visualize the results (S1 Fig We first explored the community-wide effects of TPI on species composition using data of our first monitoring year. Second, we evaluated floristic shifts as a function of time and topographic variation; thereby plot scale species composition was modeled as a function of time, TPI and their interaction. Finally, we tested for differences in species composition between dead and recruited trees, modeling species composition as a function of both demographic groups, TPI, and an interaction term.

We also calculated annual turnover rates for each plot which was estimated as the average of the annual percentage of recruitment, and annual percentage of mortality [83].

#### Community climate indices and trait composition

We used repeated measures linear mixed models to evaluate how trait composition measured as community-weighted moments (CWM, CWV, CWS, CWK) in each of our ten functional traits, and community climatic indices (CTI and CPI) changed across gradients of topography and through time. In our models, CWMs of a given functional trait or climatic index was a function of time, TPI, their interaction, and plot was included as a random factor. Similarly, we calculated repeated measures linear mixed models to evaluate the effect of topographic variation and time on the remaining three community-weighted moments (CWV, CWK, CWS). In these models, each community-weighted moment was modeled as a function of time and TPI, with plot included as a random factor. For statistical analyses we used the packages lme4 and lmerTest in R [84, 85].

We also used differences between the initial and final censuses to obtain annualized rates of change in CTI and CPI per plot (TRplot and PRplot, respectively) [18]. We calculated binomial probability distributions with TRplot, and PRplot values against the null expectation of an equal number of plots decreasing and increasing in CTI/CPI over time.

#### Community climatic indices and trait composition across demographic groups

We used linear mixed models to assess differences in trait composition (only for community-weighted means CWMs) or climatic indices across demographic groups. Our models’ fixed effects were demographic group (i.e., dead, surviving, and recruiting trees), TPI, and an interaction term; plot was included as a random factor. Then, Tukey-HSD was used to compare means among demographic groups when the interaction term was statistically significant.

## Supporting information

S1 Table Functional trait composition and community climatic indices expressed as community weighted means (CWMs) of the monitoring plots during the first year of the study. Topographic variation is expressed as a Topographic Position Index (TPI).
(DOCX)

S2 Table Repeated measures linear mixed models predicting community weighted means (CWMs) of ten functional traits and two climatic indices as a function of topography (Topographic Position Index = TPI), time, and their interaction. In the models, plot was included as a random effect. Data was collected in 18 permanent plots over eight years in southern Ecuador. Statistically significant values are presented in bold.
(DOCX)

S3 Table Linear correlations among 10 functional traits measured in 158 species in a tropical montane forest in southern Ecuador, calculated using the package cormorant (Link 2020). (DOCX)

S4 Table Linear mixed models testing for the effects of topography and time on community weighted moments (CWV, CWS, CWK) in eighteen permanent plots in southern Ecuador. Statistically significant values are marked in bold.
(DOCX)

S5 Table Linear mixed models of community-weighted means (CWM) of ten functional traits and two climatic indices as a function of demographic grouping (i.e., recruited, dead, or growing trees), plot topographic position (Topographic Position Index = TPI) and their interaction. In the models, plot was included as a random effect. Data was collected in 18 permanent plots over eight years in Southern Ecuador. Statistically significant values are presented in bold.
(DOCX)

S6 Table Methods used to assess functional traits in a set of tree species. (DOCX)

S7 Table Mean and standard error of the number of species with trait values, and percentage of plot scale basal area with trait values for the first and last year of our monitoring period. Figures are calculated for 18 20 x 20 m forest plots in Southern Ecuador.
(DOCX)

S8 Table Results of correlation analyses comparing community-weighted mean values calculated using plot scale tree basal area to plot-scale number of individual stems for trees at the beginning and the end of the monitoring period (year 1 and year 8, respectively), and for each tree demographic group. (DOCX)

S9 Table Methods used to evaluate the relevance of elevation and topography (TPI) on community floristic composition. (DOCX)

S10 Table Methods used to evaluate the relevance of elevation and topography (TPI) on community climate indices (CTI and CPI) and community weighted means. (DOCX)

S11 Table Methods and results of analyses testing for spatial autocorrelation in trait composition and community climatic indices of 18 plots in southern Ecuador. (DOCX)

S1 Fig Non-metric multidimensional scaling of tree species composition during the first year of the study, in 18 permanent plots in southern Ecuador. Topographic variation is expressed as a Topographic Position Index (TPI).
(DOCX)

S2 Fig Linear mixed models testing for the effects of topography and time on community weighted moments (CWV, CWS, CWK) in eighteen permanent plots in southern Ecuador. Statistically significant relations (p<0.05) with Topographic Position Index (TPI) are indicated by solid regression lines.
(DOCX)

S3 Fig Functional trait composition as normalized Community Weighted Means (CWM) considering three demographic groups in 18 permanent plots over eight years; CWM are a function of Topographic Position Index (TPI). Stars indicate statistically significant values at *P*≤0.05.
(DOCX)

S4 Fig A) Image of the study area showing the rugged topography, B) the distribution of the 18 permanent study plots in the San Francisco reserve and C) schematic sketch showing the slope positions and their respective Topographic Position Index values (TPI), from most negative values at valley bottoms to most positive values at ridge tops. (DOCX)

S5 Fig Correlations of soil features with Topographic Position Index (TPI) in the 18 study plots. Soil properties: Depth of organic layer (dol), pH value, concentrations of K, Mg, Ca, Al and N, C:N ratio, plant available phosphorus (Pav), nitrogen mineralization rate (Nmin) and nitrogen nitrification rate (Nnitr). Data from Wolf *et al*. (2011) as presented in Pierick et al.(2021). All correlations are statistically significant, and shadows indicate 95% confidence intervals.
(DOCX)

## References

[1] Malhi Y, Phillips OL. Tropical forests and global atmospheric change: a synthesis2004 2004-03-29 00:00:00. 549–55 p. 10.1098/rstb.2003.1449 PMC1693340 15212102

[2] Feeley KJ, Bravo-Avila C, Fadrique B, Perez TM, Zuleta D. Climate-driven changes in the composition of New World plant communities. Nature Climate Change. 2020. doi: 10.1038/s41558-020-0873-2

[3] Krishnaswamy J, John R, Joseph S. Consistent response of vegetation dynamics to recent climate change in tropical mountain regions. Global Change Biology. 2014;20(1):203–15. doi: 10.1111/gcb.12362 23966269

[4] ForestPlots.net, Blundo C, Carilla J, Grau R, Malizia A, Malizia L, et al. Taking the pulse of Earth’s tropical forests using networks of highly distributed plots. Biological Conservation. 2021:108849. doi: 10.1016/j.biocon.2020.108849

[5] Wieczynski DJ, Boyle B, Buzzard V, Duran SM, Henderson AN, Hulshof CM, et al. Climate shapes and shifts functional biodiversity in forests worldwide. Proceedings of the National Academy of Sciences. 2019;116(2):587. doi: 10.1073/pnas.1813723116 30584087 PMC6329988

[6] Šímová I, Violle C, Svenning J-C, Kattge J, Engemann K, Sandel B, et al. Spatial patterns and climate relationships of major plant traits in the New World differ between woody and herbaceous species. Journal of Biogeography. 2018;45(4):895–916. doi: 10.1111/jbi.13171

[7] Suding KN, Lavorel S., Chapin F. S., Cornelissen J. H. C., Díaz S., Garnier E., et al. Scaling environmental change through the community-level: a trait-based response-and-effect framework for plants. Global Change Biology. 2008;14:1125–40.

[8] Madani N, Kimball JS, Ballantyne AP, Affleck DLR, van Bodegom PM, Reich PB, et al. Future global productivity will be affected by plant trait response to climate. Scientific Reports. 2018;8(1):2870. doi: 10.1038/s41598-018-21172-9 29434266 PMC5809371

[9] Rahbek C, Borregaard MK, Colwell RK, Dalsgaard B, Holt BG, Morueta-Holme N, et al. Humboldt’s enigma: What causes global patterns of mountain biodiversity? Science. 2019;365(6458):1108. doi: 10.1126/science.aax0149 31515383

[10] Karger DN, Kessler M, Lehnert M, Jetz W. Limited protection and ongoing loss of tropical cloud forest biodiversity and ecosystems worldwide. Nature Ecology & Evolution. 2021. doi: 10.1038/s41559-021-01450-y 33927369

[11] Duque A, Peña MA, Cuesta F, González-Caro S, Kennedy P, Phillips OL, et al. Mature Andean forests as globally important carbon sinks and future carbon refuges. Nature Communications. 2021;12(1):2138. doi: 10.1038/s41467-021-22459-8 33837222 PMC8035207

[12] Wiens JJ. Climate-Related Local Extinctions Are Already Widespread among Plant and Animal Species. PLOS Biology. 2016;14(12):e2001104. doi: 10.1371/journal.pbio.2001104 27930674 PMC5147797

[13] Freeman BG, Lee-Yaw JA, Sunday JM, Hargreaves AL. Expanding, shifting and shrinking: The impact of global warming on species’ elevational distributions. Global Ecology and Biogeography. 2018;27(11):1268–76. doi: 10.1111/geb.12774

[14] Sheldon KS, Yang S, Tewksbury JJ. Climate change and community disassembly: impacts of warming on tropical and temperate montane community structure. Ecology Letters. 2011;14(12):1191–200. doi: 10.1111/j.1461-0248.2011.01689.x 21978234

[15] Feeley KJ, Hurtado J, Saatchi S, Silman MR, Clark DB. Compositional shifts in Costa Rican forests due to climate-driven species migrations. Global Change Biology. 2013;19:3472–80. doi: 10.1111/gcb.12300 23794172

[16] Duque A, Stevenson PR, Feeley KJ. Thermophilization of adult and juvenile tree communities in the northern tropical Andes. Proceedings of the National Academy of Sciences. 2015;112(34):10744–9. doi: 10.1073/pnas.1506570112 26261350 PMC4553780

[17] Feeley KJ, Silman MR, Bush M, Farfan W, Garcia Cabrera K, Malhi Y, et al. Upslope migration of Andean trees. Journal of Biogeography. 2011;38:783–91.

[18] Fadrique B, Báez S, Duque Á, Malizia A, Blundo C, Carilla J, et al. Widespread but heterogeneous responses of Andean forests to climate change. Nature. 2018;514:207–12. doi: 10.1038/s41586-018-0715-9 30429613

[19] Homeier J, Seeler T, Pierick K, Leuschner C. Leaf trait variation in species-rich tropical Andean forests. Scientific Reports. 2021;11(1):9993. doi: 10.1038/s41598-021-89190-8 33976239 PMC8113502

[20] Steinbauer MJ, Grytnes J-A, Jurasinski G, Kulonen A, Lenoir J, Pauli H, et al. Accelerated increase in plant species richness on mountain summits is linked to warming. Nature. 2018;556(7700):231–4. doi: 10.1038/s41586-018-0005-6 29618821

[21] Rumpf SB, Hülber K, Klonner G, Moser D, Schütz M, Wessely J, et al. Range dynamics of mountain plants decrease with elevation. Proceedings of the National Academy of Sciences. 2018;115(8):1848. doi: 10.1073/pnas.1713936115 29378939 PMC5828587

[22] McLaughlin BC, Ackerly DD, Klos PZ, Natali J, Dawson TE, Thompson SE. Hydrologic refugia, plants, and climate change. Global Change Biology. 2017;23(8):2941–61. doi: 10.1111/gcb.13629 28318131

[23] Crimmins SM, Dobrowski SZ, Greenberg JA, Abatzoglou JT, Mynsberge AR. Changes in Climatic Water Balance Drive Downhill Shifts in Plant Species’ Optimum Elevations. Science. 2011;331(6015):324. doi: 10.1126/science.1199040 21252344

[24] McCain CM, Colwell RK. Assessing the threat to montane biodiversity from discordant shifts in temperature and precipitation in a changing climate. Ecology Letters. 2011;14(12):1236–45. doi: 10.1111/j.1461-0248.2011.01695.x 21981631

[25] Urrutia R, Vuille M. Climate change projections for the tropical Andes using a regional climate model: Temperature and precipitation simulations for the end of the 21st century. Journal of Geophysical Research: Atmospheres. 2009;114(D2). doi: 10.1029/2008JD011021

[26] Vuille M, Franquist E, Garreaud R, Lavado Casimiro aWS, Cáceres B. Impact of the global warming hiatus on Andean temperature. Journal of Geophisical Research. 2015;120:3745–57.

[27] Rehm E, Feeley KJ. Many species risk mountain top extinction long before they reach the top. Frontiers of Biogeography. 2016;8:e27788, 2016. doi: 10.21425/F5FBG27788

[28] Jucker T, Bongalov B, Burslem DFRP, Nilus R, Dalponte M, Lewis SL, et al. Topography shapes the structure, composition and function of tropical forest landscapes. Ecology Letters. 2018;21(7):989–1000. doi: 10.1111/ele.12964 29659115 PMC6849614

[29] Werner FA, Homeier J. Is tropical montane forest heterogeneity promoted by a resource-driven feedback cycle? Evidence from nutrient relations, herbivory and litter decomposition along a topographical gradient. Functional Ecology. 2015;29(3):430–40. doi: 10.1111/1365-2435.12351

[30] Homeier J, Breckle S-W, Günter S, Rollenbeck RT, Leuschner C. Tree diversity, forest structure and productivity along altitudinal and topographical gradients in a species-rich Ecuadorian montane rain forest. Biotropica. 2010;42(2):140–8. doi: 10.1111/j.1744-7429.2009.00547.x

[31] Fortunel C, Lasky JR, Uriarte M, Valencia R, Wright SJ, Garwood NC, et al. Topography and neighborhood crowding can interact to shape species growth and distribution in a diverse Amazonian forest. Ecology. 2018;99:2272–83. doi: 10.1002/ecy.2441 29975420

[32] Moeslund JE, Arge L, Bøcher PK, Dalgaard T, Svenning J-C. Topography as a driver of local terrestrial vascular plant diversity patterns. Nordic Journal of Botany. 2013;31(2):129–44. doi: 10.1111/j.1756-1051.2013.00082.x

[33] Mage SM, Porder S. Parent Material and Topography Determine Soil Phosphorus Status in the Luquillo Mountains of Puerto Rico. Ecosystems. 2013;16(2):284–94. doi: 10.1007/s10021-012-9612-5

[34] Allié E, Pélissier R, Engel J, Petronelli P, Freycon V, Deblauwe V, et al. Pervasive Local-Scale Tree-Soil Habitat Association in a Tropical Forest Community. PLOS ONE. 2015;10(11):e0141488. doi: 10.1371/journal.pone.0141488 26535570 PMC4633048

[35] Lippok D, Beck SG, Renison D, Hensen I, Apaza AE, Schleuning M. Topography and edge effects are more important than elevation as drivers of vegetation patterns in a neotropical montane forest. Journal of Vegetation Science. 2014;25(3):724–33. doi: 10.1111/jvs.12132

[36] Davis FW, Synes NW, Fricker GA, McCullough IM, Serra-Diaz JM, Franklin J, et al. LiDAR-derived topography and forest structure predict fine-scale variation in daily surface temperatures in oak savanna and conifer forest landscapes. Agricultural and Forest Meteorology. 2019;269–270:192–202. doi: 10.1016/j.agrformet.2019.02.015

[37] Ackerly DD, Loarie SR, Cornwell WK, Weiss SB, Hamilton H, Branciforte R, et al. The geography of climate change: implications for conservation biogeography. Diversity and Distributions. 2010;16(3):476–87. doi: 10.1111/j.1472-4642.2010.00654.x

[38] Méndez-Toribio M, Meave JA, Zermeño-Hernández I, Ibarra-Manríquez G. Effects of slope aspect and topographic position on environmental variables, disturbance regime and tree community attributes in a seasonal tropical dry forest. Journal of Vegetation Science. 2016;27(6):1094–103. doi: 10.1111/jvs.12455

[39] Méndez-Toribio M, Ibarra-Manríquez G, Navarrete-Segueda A, Paz H. Topographic position, but not slope aspect, drives the dominance of functional strategies of tropical dry forest trees. Environmental Research Letters. 2017;12:085002.

[40] Kraft NJB, Valencia R, Ackerly DD. Functional Traits and Niche-Based Tree Community Assembly in an Amazonian Forest. Science. 2008;322(5901):580. doi: 10.1126/science.1160662 18948539

[41] Bueno de Mesquita CP, Tillmann LS, Bernard CD, Rosemond KC, Molotch NP, Suding KN. Topographic heterogeneity explains patterns of vegetation response to climate change (1972–2008) across a mountain landscape, Niwot Ridge, Colorado. Arctic, Antarctic, and Alpine Research. 2018;50(1):e1504492. doi: 10.1080/15230430.2018.1504492

[42] He S, Zhong Y, Sun Y, Su Z, Jia X, Hu Y, et al. Topography-associated thermal gradient predicts warming effects on woody plant structural diversity in a subtropical forest. Scientific Reports. 2017;7:40387. doi: 10.1038/srep40387 https://www.nature.com/articles/srep40387 - supplementary-information. 28067326 PMC5220297

[43] Lenoir J, Hattab T, Pierre G. Climatic microrefugia under anthropogenic climate change: implications for species redistribution. Ecography. 2017;40(2):253–66. doi: 10.1111/ecog.02788

[44] Lembrechts JJ, Nijs I, Lenoir J. Incorporating microclimate into species distribution models. Ecography. 2019;42(7):1267–79. doi: 10.1111/ecog.03947

[45] Maclean IMD, Suggitt AJ, Wilson RJ, Duffy JP, Bennie JJ. Fine-scale climate change: modelling spatial variation in biologically meaningful rates of warming. Global Change Biology. 2017;23(1):256–68. doi: 10.1111/gcb.13343 27151406

[46] Reich PB. The world-wide ‘fast–slow’ plant economics spectrum: a traits manifesto. Journal of Ecology. 2014;102:275–301.

[47] van der Sande MT, Arets EJMM, Peña-Claros M, de Avila AL, Roopsind A, Mazzei L, et al. Old-growth Neotropical forests are shifting in species and trait composition. Ecological Monographs. 2016;86:228–43.

[48] Enquist BJ, Norberg J, Bonser SP, Violle C, Webb CT, Henderson A, et al. Scaling from Traits to Ecosystems: Developing a General Trait Driver Theory via Integrating Trait-Based and Metabolic Scaling Theories. In: Samraat Pawar GW, Anthony ID, editors. Advances in Ecological Research. Volume 52: Academic Press; 2015. p. 249–318.

[49] Valencia R, Condit R, Muller-Landau HC, Hernandez C, Navarrete H. Dissecting biomass dynamics in a large Amazonian forest plot. Journal of Tropical Ecology. 2009;25(5):473–82. doi: 10.1017/S0266467409990095

[50] Pierick K, Leuschner C, Homeier J. Topography as a factor driving small-scale variation in tree fine root traits and root functional diversity in a species-rich tropical montane forest. New Phytologist. 2021;230(1):129–38. doi: 10.1111/nph.17136 33278844

[51] Homeier J, Werner FA, Gradstein R, Breckle S-W, Richter M. Potential vegetation and floristic composition of Andean forests in South Ecuador, with a focus on the RBSF. In: Beck E BJ, Kottke I, Makeschin F, Mosandl R, editor. Gradients in a Tropical Mountain Ecosystem of Ecuador. Ecological Studies. 198. Berlin, Heidelberg, New York: Springer Verlag; 2008. p. 87–100. doi: 10.1016/j.micpath.2008.08.005 18848980

[52] Sonnier G, Johnson SE, Amatangelo KL, Rogers DA, Waller DM. Is taxonomic homogenization linked to functional homogenization in temperate forests? Global Ecology and Biogeography. 2014;23(8):894–902. doi: 10.1111/geb.12164

[53] White HJ, Montgomery WI, Storchová L, Hořák D, Lennon JJ. Does functional homogenization accompany taxonomic homogenization of British birds and how do biotic factors and climate affect these processes? Ecology and Evolution. 2018;8(15):7365–77. doi: 10.1002/ece3.4267 30151156 PMC6106174

[54] Robroek BJM, Jassey VEJ, Payne RJ, Martí M, Bragazza L, Bleeker A, et al. Taxonomic and functional turnover are decoupled in European peat bogs. Nature Communications. 2017;8(1):1161. doi: 10.1038/s41467-017-01350-5 29079831 PMC5660083

[55] Ruiz-Benito P, Ratcliffe S, Jump AS, Gómez-Aparicio L, Madrigal-González J, Wirth C, et al. Functional diversity underlies demographic responses to environmental variation in European forests. Global Ecology and Biogeography. 2017;26(2):128–41. doi: 10.1111/geb.12515

[56] Peña MA, Feeley KJ, Duque A. Effects of endogenous and exogenous processes on aboveground biomass stocks and dynamics in Andean forests. Plant Ecology. 2018;219(12):1481–92. doi: 10.1007/s11258-018-0895-2

[57] Báez S, Malizia A, Carilla J, Blundo C, Aguilar M, Aguirre N, et al. Large-Scale Patterns of Turnover and Basal Area Change in Andean Forests. PLoS ONE. 2015;10(5):e0126594. doi: 10.1371/journal.pone.0126594 25973977 PMC4431807

[58] Wilcke W, Velescu A, Leimer S, Bigalke M, Boy J, Valarezo C. Temporal Trends of Phosphorus Cycling in a Tropical Montane Forest in Ecuador During 14 Years. Journal of Geophysical Research: Biogeosciences. 2019;124(5):1370–86. doi: 10.1029/2018JG004942

[59] Wilcke W, Leimer S, Peters T, Emck P, Rollenbeck R, Trachte K, et al. The nitrogen cycle of tropical montane forest in Ecuador turns inorganic under environmental change. Global Biogeochemical Cycles. 2013;27(4):1194–204. doi: 10.1002/2012GB004471

[60] Báez S, Homeier J. Functional traits determine tree growth and ecosystem productivity of a tropical montane forest: Insights from a long‐term nutrient manipulation experiment. Global Change Biology. 2018;24(1):399–409. doi: 10.1111/gcb.13905 28921844

[61] Sanchez-Martinez P, Martínez-Vilalta J, Dexter KG, Segovia RA, Mencuccini M. Adaptation and coordinated evolution of plant hydraulic traits. Ecology Letters. 2020;23(11):1599–610. doi: 10.1111/ele.13584 32808458

[62] Peters T, Drobnik T, Meyer H, Rankl M, Richter M, Rollenbeck R, et al. Environmental Changes Affecting the Andes of Ecuador. In: Jörg Bendix EB, Achim Bräuning, Franz Makeschin, Reinhard Mosandl, Stefan Scheu, Wolfgang Wilcke, editor. Ecosystem Services, Biodiversity and Environmental Change in a Tropical Mountain Ecosystem of South Ecuador. Berlin Heidelberg: Springer-Verlag; 2013. p. 19–29.

[63] Rohde R, Muller R, Jacobsen R, Perlmutter S, Rosenfeld A, Wurtele J, et al. Berkeley Earth. Geoinformatics & Geostatistics: An Overview. 2013;1. doi: 10.4172/2327-4581.1000103

[64] Loarie SR, Duffy PB, Hamilton H, Asner GP, Field CB, Ackerly DD. The velocity of climate change. Nature. 2009;462:1052. doi: 10.1038/nature08649 https://www.nature.com/articles/nature08649 - supplementary-information. 20033047

[65] De Frenne P, Lenoir J, Luoto M, Scheffers BR, Zellweger F, Aalto J, et al. Forest microclimates and climate change: Importance, drivers and future research agenda. Global Change Biology. 2021;27(11):2279–97. doi: 10.1111/gcb.15569 33725415

[66] Brown CD, Vellend M. Non-climatic constraints on upper elevational plant range expansion under climate change. Proceedings of the Royal Society B: Biological Sciences. 2014;281(1794):20141779. doi: 10.1098/rspb.2014.1779 25253462 PMC4211457

[67] Schweiger AH, Beierkuhnlein C. Scale dependence of temperature as an abiotic driver of species’ distributions. Global Ecology and Biogeography. 2016;25(8):1013–21. doi: 10.1111/geb.12463

[68] Lenoir J, Svenning JC. Climate-related range shifts–a global multidimensional synthesis and new research directions. Ecography. 2015;38(1):15–28. doi: 10.1111/ecog.00967

[69] Wolf K, Veldkamp E, Homeier J, Martinson GO. Nitrogen availability links forest productivity, soil nitrous oxide and nitric oxide fluxes of a tropical montane forest in southern Ecuador. Global Biogeochemical Cycles. 2011;25(4):GB4009. doi: 10.1029/2010GB003876

[70] Wilcke W, Oelmann Y, Schmitt A, Valarezo C, Zech W, Homeier J. Soil properties and tree growth along an altitudinal transect in Ecuadorian tropical montane forest. Journal of Plant Nutrition and Soil Science. 2008;171(2):220–30. doi: 10.1002/jpln.200625210

[71] Álava P, Silva B, Schulz M, Rollenbeck RT, Bendix J. Evapotranspiration estimates for two tropical mountain forest using high spatial resolution satellite data. International Journal of Remote Sensing. 2021;42(8):2940–62. doi: 10.1080/01431161.2020.1864058

[72] Weiss A, editor Topographic position and landforms analysis. Poster presentation. ESRI user conference; 2001; San Diego, CA.

[73] Ungerechts L. DEM 10m (triangulated from aerial photo—b/w): DFG-FOR816dw; 2010 [cited 2013 2013-05-27].

[74] Link R. Calculation of the Topographical Position Index, slope and aspect for the MATRIX plots (Loja/Zamora province, Southern Ecuador). 2018 [cited 2017 1.Nov].

[75] Enquist BJ, Condit R, Peet RK, Schildhauer M, Thiers B. The botanical information and ecology network (BIEN): cyberinfrastructure for an integrated botanical information network to investigate the ecological impacts of global climate change on plant biodiversity Tuscon, Arizona, USA: The iPlant Collaborative; 2009. www.iplantcollaborative.org/sites/default/files/BIEN_White_Paper.pdf.

[76] Romoleroux K, Pérez A, León-Yánez S, Quintana C, Navarrete H, Muriel P, et al. Base de datos del Herbario QCA Quito, Ecuador: Pontificia Universidad Católica del Ecuador; 2018 [cited 2018 July/15]. Version 1.0:[<https://bioweb.bio/portal/>

[77] Karger DN, Conrad O, Böhner J, Kawohl T, Kreft H, Soria-Auza RW, et al. Climatologies at high resolution for the earth’s land surface areas. Scientific Data. 2017;4:170122. doi: 10.1038/sdata.2017.122 28872642 PMC5584396

[78] Muscarella R, Lohbeck M, Martínez-Ramos M, Poorter L, Rodríguez-Velázquez JE, van Breugel M, et al. Demographic drivers of functional composition dynamics. Ecology. 2017;98(11):2743–50. doi: 10.1002/ecy.1990 28833040

[79] Symonds ME, Moussalli A. A brief guide to model selection, multimodel inference and model averaging in behavioural ecology using Akaike’s information criterion. Behav Ecol Sociobiol. 2011;65(1):13–21. doi: 10.1007/s00265-010-1037-6

[80] Dormann C, McPherson J, Araújo M, Bivand R, Bolliger J, Carl G, et al. Methods to account for spatial autocorrelation in the analysis of species distributional data: a review. Ecography. 2007;30(5):609–28. doi: 10.1111/j.2007.0906-7590.05171.x

[81] Oksanen J, Blanchet FG, Friendly M, Kindt R, Legendre P, McGlinn D, et al. vegan: Community Ecology Package. Ordination methods, diversity analysis and other functions for community and vegetation ecologists2016.

[82] Team RC. R: a language and environment for statistical computing. R Foundation for Statistical Computing. R Foundation for Statistical Computing. Vienna, Austria2020.

[83] Stephenson NL, van Mantgem PJ. Forest turnover rates follow global and regional patterns of productivity. Ecology Letters. 2005;8(5):524–31. doi: 10.1111/j.1461-0248.2005.00746.x 21352456

[84] Kuznetsova A, Brockhoff P, Christensen R. lmerTest Package: Tests in Linear Mixed Effects Models. Journal of Statistical Software. 2017;82:1–26. doi: 10.18637/jss.v082.i13

[85] Bates D, Maechler M, Bolker B, Walker S. Fitting Linear Mixed-Effects Models Using lme4. Journal of Statistical Software. 2015;67:1–48.

