# Supplementary material for: Changes in tree functional composition across topographic gradients and through time in a tropical montane forest
Source: PLoS One. 2022 Apr 20;17(4):e0263508. doi: 10.1371/journal.pone.0263508 (PMC9020722; doi:10.1371/journal.pone.0263508)
Supplement: S1 Table — Topographic variation is expressed as a Topographic Position Index (TPI). (DOCX) [file pone.0263508.s001.docx]

**S1 Table.** **Functional trait composition and community climatic indices expressed as community weighted means (CWMs) of the monitoring plots during the first year of the study.** Topographic variation is expressed as a Topographic Position Index (TPI).

| **Plot** | **TPI** | **Elevation (m)** | **Bark thickness** | **Foliar N** | **Foliar P** | **Leaf area** | **Leaf toughness** | **Stem conductivity (KS)** | **Specific leaf area** | **Vessel density** | **Vessel diameter** | **Wood density** | **Community Thermal Index (CTI)** | **Community Precipitation Index (CPI)** |
| --- | --- | --- | --- | --- | --- | --- | --- | --- | --- | --- | --- | --- | --- | --- |
| 4 | -1.11 | 1913 | 0.49 | 25.71 | 1.38 | 121.92 | 0.65 | 42.85 | 126.61 | 103.83 | 8.96 | 0.46 | 19.18 | 1919.65 |
| 10 | -0.8 | 1927 | 0.52 | 25.44 | 1.82 | 144.51 | 0.58 | 34.31 | 141.95 | 102.08 | 9.70 | 0.40 | 19.45 | 2076.87 |
| 2 | -0.54 | 1993 | 0.47 | 21.50 | 1.20 | 128.07 | 1.04 | 60.61 | 94.74 | 126.24 | 7.59 | 0.54 | 21.12 | 2352.43 |
| 5 | -0.49 | 1954 | 0.49 | 21.97 | 1.32 | 437.82 | 0.72 | 51.24 | 92.11 | 129.43 | 6.70 | 0.41 | 18.55 | 1956.92 |
| 12 | -0.37 | 2020 | 0.50 | 18.62 | 0.93 | 193.00 | 0.82 | 40.21 | 76.85 | 107.64 | 12.03 | 0.51 | 19.48 | 1952.48 |
| 6 | -0.35 | 1933 | 0.65 | 18.23 | 0.88 | 130.77 | 0.88 | 54.27 | 83.33 | 130.52 | 34.20 | 0.48 | 19.80 | 1925.75 |
| 3 | -0.33 | 2020 | 0.47 | 21.65 | 1.25 | 77.45 | 0.98 | 47.54 | 99.34 | 113.14 | 11.37 | 0.53 | 20.43 | 2301.42 |
| 11 | -0.29 | 1971 | 0.57 | 23.95 | 1.49 | 176.52 | 0.66 | 33.93 | 116.28 | 95.71 | 10.33 | 0.48 | 20.89 | 2196.11 |
| 8 | -0.3 | 1950 | 0.54 | 22.36 | 1.23 | 139.07 | 0.86 | 25.76 | 102.13 | 95.49 | 11.04 | 0.52 | 19.82 | 2173.94 |
| 7 | -0.02 | 2034 | 0.51 | 19.55 | 0.85 | 84.90 | 1.07 | 30.86 | 74.30 | 94.93 | 13.00 | 0.55 | 19.24 | 2115.41 |
| 1 | -0.01 | 2039 | 0.53 | 17.14 | 0.96 | 73.33 | 1.17 | 45.86 | 74.78 | 110.32 | 9.25 | 0.55 | 18.35 | 2214.64 |
| 9 | 0.14 | 2027 | 0.47 | 16.57 | 0.85 | 213.13 | 0.98 | 40.43 | 68.73 | 108.80 | 9.80 | 0.53 | 19.28 | 1816.37 |
| 15 | 0.66 | 2089 | 0.45 | 14.18 | 0.61 | 56.01 | 1.30 | 30.98 | 56.37 | 89.30 | 54.76 | 0.56 | 16.49 | 1867.67 |
| 16 | 0.76 | 2063 | 0.57 | 15.20 | 0.62 | 46.56 | 1.59 | 30.23 | 54.34 | 86.00 | 43.13 | 0.59 | 16.70 | 1835.10 |
| 14 | 1.1 | 2026 | 0.39 | 14.77 | 0.67 | 45.68 | 1.33 | 33.13 | 57.59 | 99.87 | 23.87 | 0.60 | 17.72 | 1814.05 |
| 17 | 1.14 | 2054 | 0.48 | 15.57 | 0.63 | 34.57 | 1.14 | 33.62 | 59.84 | 91.52 | 18.36 | 0.58 | 17.09 | 1928.84 |
| 13 | 1.15 | 2002 | 0.41 | 13.18 | 0.61 | 45.57 | 1.21 | 31.52 | 54.21 | 94.61 | 16.21 | 0.60 | 17.05 | 1818.59 |
| 18 | 1.21 | 2039 | 0.43 | 14.79 | 0.68 | 33.07 | 1.42 | 36.38 | 54.35 | 98.98 | 39.40 | 0.59 | 16.83 | 1824.23 |
| Mean | 0.09 | 2003 | 0.50 | 18.91 | 1.00 | 121.22 | 1.02 | 39.09 | 82.66 | 104.36 | 18.87 | 0.53 | 18.75 | 2005.03 |
| SE | 0.17 | 11.98 | 0.01 | 0.95 | 0.08 | 22.86 | 0.07 | 2.25 | 6.28 | 3.16 | 3.37 | 0.01 | 0.35 | 42.26 |
