# Supplementary material for: Changes in tree functional composition across topographic gradients and through time in a tropical montane forest
Source: PLoS One. 2022 Apr 20;17(4):e0263508. doi: 10.1371/journal.pone.0263508 (PMC9020722; doi:10.1371/journal.pone.0263508)
Supplement: S2 Table — In the models, plot was included as a random effect. Data was collected in 18 permanent plots over eight years in southern Ecuador. Statistically significant values are presented in bold. (DOCX) [file pone.0263508.s002.docx]

**S2 Table. Repeated measures linear mixed models predicting community weighted means (CWMs) of ten functional traits and two climatic indices as a function of topography (Topographic Position Index= TPI), time, and their interaction.** In the models, plot was included as a random effect. Data was collected in 18 permanent plots over eight years in southern Ecuador. Statistically significant values are presented in bold.

| **Functional trait /**  **Community climatic index** | **TPI** | | | **Time** | | | **TPI x time** | | | **Random effects (Variance)** | |
| --- | --- | --- | --- | --- | --- | --- | --- | --- | --- | --- | --- |
|  | Est. | t | P | Est. | t | P | Est. | t | P | Plot | Residual |
| Bark thickness | -0.17 | -2.02 | 0.056 | 0.01 | 1.34 | 0.199 | -0.01 | -1.06 | 0.307 | 0.05 | 0.01 |
| Leaf area (LA) | -0.35 | -2.70 | **0.015** | -0.01 | -1.64 | 0.120 | 0.01 | 1.52 | 0.148 | 0.15 | 0.01 |
| Leaf toughness | 0.77 | 6.24 | **<0.001** | -0.01 | -1.55 | 0.140 | 0.00 | 0.11 | 0.916 | 0.09 | 0.03 |
| Foliar nitrogen | -0.82 | -8.30 | **<0.001** | 0.01 | 1.74 | 0.102 | -0.01 | -1.28 | 0.219 | 0.08 | 0.01 |
| Foliar phosphorus | -0.79 | -5.53 | **<0.001** | 0.00 | 0.58 | 0.571 | 0.00 | -0.26 | 0.795 | 0.15 | 0.03 |
| Sapwood-specific conductivity (KS) | -0.22 | -1.85 | 0.077 | 0.00 | 0.30 | 0.770 | -0.01 | -1.00 | 0.333 | 0.01 | 0.01 |
| Specific leaf area (SLA) | -0.81 | -6.34 | **<0.001** | 0.01 | 2.03 | 0.060 | -0.01 | -1.13 | 0.274 | 0.13 | 0.02 |
| Vessel density | -0.34 | -2.52 | **0.021** | 0.00 | -0.42 | 0.680 | 0.00 | 0.20 | 0.847 | 0.08 | 0.01 |
| Vessel diameter | 0.16 | 3.13 | **0.004** | 0.00 | -0.89 | 0.387 | -0.01 | -1.05 | 0.309 | 0.15 | 0.01 |
| Wood density [WSG] | 0.61 | 6.16 | **<0.001** | 0.00 | -0.16 | 0.877 | 0.00 | 0.62 | 0.547 | 0.78 | 0.11 |
| Community temperature index (CTI) | -1.63 | -5.12 | **<0.001** | -0.02 | -1.39 | 0.182 | 0.03 | 1.40 | 0.182 | 0.12 | 0.01 |
| Community precipitation index (CPI) | -142.25 | -2.76 | **0.013** | -1.75 | -1.30 | 0.211 | 3.64 | 1.93 | 0.070 | 23147.8 | 792.5 |
