# Supplementary material for: Changes in tree functional composition across topographic gradients and through time in a tropical montane forest
Source: PLoS One. 2022 Apr 20;17(4):e0263508. doi: 10.1371/journal.pone.0263508 (PMC9020722; doi:10.1371/journal.pone.0263508)
Supplement: S3 Table — (DOCX) [file pone.0263508.s003.docx]

**S3Table. Linear correlations among 10 functional traits measured in 158 species in a tropical montane forest in southern Ecuador, calculated using the package cormorant (Link 2020).**

**
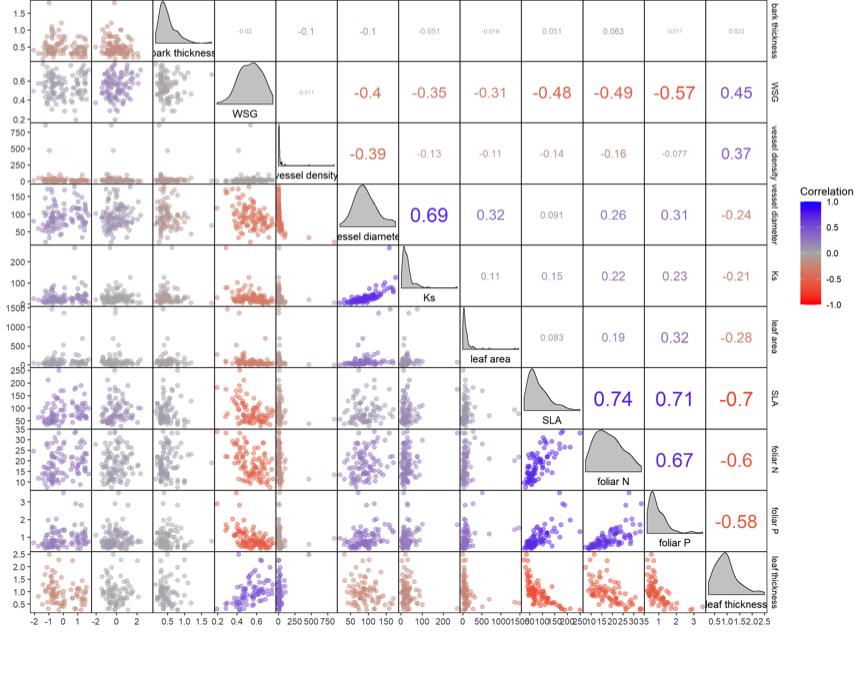
**

**Reference:** Roman M. Link (2020). corrmorant: Flexible Correlation Matrices Based on 'ggplot2'. R package version 0.0.0.9007. <http://github.com/r-link/corrmorant>
