# Supplementary material for: Changes in tree functional composition across topographic gradients and through time in a tropical montane forest
Source: PLoS One. 2022 Apr 20;17(4):e0263508. doi: 10.1371/journal.pone.0263508 (PMC9020722; doi:10.1371/journal.pone.0263508)
Supplement: S4 Table — Statistically significant values are marked in bold. (DOCX) [file pone.0263508.s004.docx]

**S4 Table.** **Linear mixed models testing for the effects of topography and time on community weighted moments (CWV, CWS, CWK) in eighteen permanent plots in Southern Ecuador.** Statistically significant values are marked in bold.

| **Community weighted moment** | **Trait** | **Fixed effects** | | | | | | **Random effects** | |
| --- | --- | --- | --- | --- | --- | --- | --- | --- | --- |
|  |  | **Term** | **Est.** | **SE** | **F** | **df** | **P** | **Term** | **Est.** |
| CWVariance | Bark thickness | Int. | 0.58 | 0.09 | 6.53 | 16.83 | 0.000 | Plot | 0.37 |
|  |  | TPI | 0.03 | 0.12 | 0.28 | 16.00 | 0.781 | Res. | 0.09 |
|  |  | Time | 0.01 | 0.03 | 0.29 | 17.00 | 0.776 |  |  |
|  | Foliar N | Int. | 0.52 | 0.05 | 9.69 | 16.35 | 0.000 | Plot | 0.22 |
|  |  | TPI | -0.10 | 0.07 | -1.27 | 16.00 | 0.221 | Res. | 0.03 |
|  |  | Time | 0.01 | 0.01 | 0.87 | 17.00 | 0.398 |  |  |
|  | Foliar P | Int. | 0.71 | 0.14 | 4.97 | 17.39 | 0.000 | Plot | 0.58 |
|  |  | **TPI** | -0.68 | 0.19 | -3.51 | 16.00 | **0.003** | Res. | 0.17 |
|  |  | Time | 0.00 | 0.06 | 0.03 | 17.00 | 0.974 |  |  |
|  | Leaf area [LA] | Int. | 1.15 | 0.44 | 2.59 | 17.31 | 0.019 | Plot | 1.79 |
|  |  | TPI | -0.80 | 0.61 | -1.32 | 16.00 | 0.204 | Res. | 0.53 |
|  |  | Time | -0.16 | 0.18 | -0.94 | 17.00 | 0.361 |  |  |
|  | Leaf toughness | Int. | 0.70 | 0.06 | 11.09 | 22.01 | 0.000 | Plot | 0.22 |
|  |  | **TPI** | 0.29 | 0.08 | 3.59 | 16.00 | **0.002** | Res. | 0.15 |
|  |  | Time | -0.04 | 0.05 | -0.84 | 17.00 | 0.413 |  |  |
|  | Sapwood-specific conductivity [KS] | Int. | 1.09 | 0.41 | 2.66 | 23.08 | 0.014 | Plot | 1.36 |
|  |  | **TPI** | -1.52 | 0.52 | -2.95 | 16.00 | **0.009** | Res. | 1.07 |
|  |  | Time | 0.30 | 0.36 | 0.85 | 17.00 | 0.405 |  |  |
|  | Specific leaf area [SLA] | Int. | 0.64 | 0.09 | 7.14 | 17.40 | 0.000 | Plot | 0.36 |
|  |  | **TPI** | -0.50 | 0.12 | -4.10 | 16.00 | **0.001** | Res. | 0.11 |
|  |  | Time | 0.04 | 0.04 | 1.18 | 17.00 | 0.256 |  |  |
|  | Vessel density | Int. | 0.69 | 0.30 | 2.31 | 19.53 | 0.032 | Plot | 1.12 |
|  |  | TPI | 0.36 | 0.39 | 0.91 | 16.00 | 0.376 | Res. | 0.57 |
|  |  | Time | -0.18 | 0.19 | -0.96 | 17.00 | 0.349 |  |  |
|  | Vessel diameter | Int. | 0.94 | 0.09 | 10.92 | 18.94 | 0.000 | Plot | 0.33 |
|  |  | **TPI** | -0.27 | 0.11 | -2.36 | 16.00 | **0.031** | Res. | 0.15 |
|  |  | Time | -0.04 | 0.05 | -0.73 | 17.00 | 0.475 |  |  |
|  | Wood density [WSG] | Int. | 0.62 | 0.07 | 8.85 | 17.15 | 0.000 | Plot | 0.29 |
|  |  | TPI | -0.14 | 0.10 | -1.46 | 16.00 | 0.163 | Res. | 0.08 |
|  |  | Time | 0.01 | 0.03 | 0.23 | 17.00 | 0.818 |  |  |
|  | Community Thermal Index (CTI) | Int. | 0.73 | 0.07 | 10.87 | 21.25 | 0.000 | Plot | 0.24 |
|  |  | TPI | -0.09 | 0.09 | -1.01 | 16.00 | 0.326 | Res. | 0.15 |
|  |  | Time | 0.00 | 0.05 | 0.04 | 17.00 | 0.971 |  |  |
|  | Community Precipitation Index (CTI) | Int. | 0.49 | 0.06 | 8.94 | 16.99 | 0.000 | Plot | 0.23 |
|  |  | TPI | -0.01 | 0.08 | -0.13 | 16.00 | 0.900 | Res. | 0.06 |
|  |  | Time | 0.01 | 0.02 | 0.72 | 17.00 | 0.482 |  |  |
| CWSkeweness | Bark thickness | Int. | 1.25 | 0.23 | 5.53 | 18.00 | 0.000 | Plot | 0.89 |
|  |  | TPI | 0.47 | 0.31 | 1.55 | 16.00 | 0.140 | Res. | 0.33 |
|  |  | Time | -0.22 | 0.11 | -1.96 | 17.00 | 0.066 |  |  |
|  | Foliar N | Int. | 0.57 | 0.14 | 4.15 | 17.44 | 0.001 | Plot | 0.55 |
|  |  | TPI | 0.33 | 0.19 | 1.77 | 16.00 | 0.096 | Res. | 0.17 |
|  |  | Time | -0.09 | 0.06 | -1.50 | 17.00 | 0.152 |  |  |
|  | Foliar P | Int. | 1.40 | 0.29 | 4.75 | 17.54 | 0.000 | Plot | 1.18 |
|  |  | **TPI** | -0.88 | 0.40 | -2.20 | 16.00 | **0.043** | Res. | 0.38 |
|  |  | Time | -0.04 | 0.13 | -0.35 | 17.00 | 0.730 |  |  |
|  | Leaf area | Int. | 2.63 | 0.35 | 7.63 | 16.92 | 0.000 | Plot | 1.12 |
|  |  | TPI | 0.42 | 0.47 | 0.89 | 16.00 | 0.388 | Res. | 0.47 |
|  |  | Time | 0.00 | 0.12 | 0.03 | 17.00 | 0.974 |  |  |
|  | Leaf toughness | Int. | 0.33 | 0.23 | 1.42 | 17.96 | 0.174 | Plot | 0.92 |
|  |  | TPI | 0.14 | 0.31 | 0.46 | 16.00 | 0.655 | Res. | 0.34 |
|  |  | Time | -0.04 | 0.11 | -0.36 | 17.00 | 0.726 |  |  |
|  | Sapwood-specific conductivity [KS] | Int. | 0.92 | 0.29 | 3.22 | 18.58 | 0.005 | Plot | 1.41 |
|  |  | TPI | -0.72 | 0.39 | -1.86 | 16.00 | 0.081 | Res. | 0.35 |
|  |  | Time | -0.23 | 0.16 | -1.48 | 17.00 | 0.156 |  |  |
|  | Specific leaf area [SLA] | Int. | 1.26 | 0.20 | 6.48 | 17.86 | 0.000 | Plot | 0.78 |
|  |  | TPI | 0.16 | 0.26 | 0.61 | 16.00 | 0.549 | Res. | 0.27 |
|  |  | Time | -0.04 | 0.09 | -0.42 | 17.00 | 0.678 |  |  |
|  | Vessel density | Int. | 4.95 | 1.21 | 4.10 | 21.86 | 0.000 | Plot | 4.20 |
|  |  | TPI | 1.31 | 1.55 | 0.85 | 16.00 | 0.410 | Res. | 2.88 |
|  |  | Time | 1.12 | 0.96 | 1.17 | 17.00 | 0.258 |  |  |
|  | Vessel diameter | Int. | -0.09 | 0.16 | -0.55 | 17.38 | 0.586 | Plot | 0.64 |
|  |  | TPI | -0.07 | 0.22 | -0.30 | 16.00 | 0.768 | Res. | 0.19 |
|  |  | Time | 0.12 | 0.06 | 1.79 | 17.00 | 0.092 |  |  |
|  | Wood density [WSG] | Int. | 0.05 | 0.17 | 0.33 | 20.35 | 0.745 | Plot | 0.61 |
|  |  | TPI | 0.05 | 0.22 | 0.23 | 16.00 | 0.819 | Res. | 0.35 |
|  |  | Time | 0.06 | 0.12 | 0.55 | 17.00 | 0.588 |  |  |
|  | Community Thermal Index (CTI) | Int. | 0.03 | 0.21 | 0.13 | 17.14 | 0.901 | Plot | 0.85 |
|  |  | **TPI** | 1.15 | 0.29 | 4.00 | 16.00 | **0.001** | Res. | 0.23 |
|  |  | Time | 0.04 | 0.08 | 0.56 | 17.00 | 0.582 |  |  |
|  | Community Precipitation Index (CPI) | Int. | -0.25 | 0.24 | -1.05 | 16.74 | 0.308 | Plot | 0.99 |
|  |  | **TPI** | 0.73 | 0.33 | 2.19 | 16.00 | **0.044** | Res. | 0.22 |
| CWKurtosis | Bark thickness | Time | 0.05 | 0.07 | 0.68 | 17.00 | 0.505 |  |  |
|  |  | Int. | -0.01 | 0.88 | -0.01 | 17.31 | 0.995 | Plot | 3.56 |
|  |  | TPI | 0.89 | 1.20 | 0.74 | 16.00 | 0.469 | Res. | 1.05 |
|  |  | Time | -0.71 | 0.35 | -2.05 | 17.00 | 0.056 |  |  |
|  | Foliar N | Int. | -2.46 | 0.59 | -4.17 | 17.58 | 0.001 | Plot | 2.36 |
|  |  | TPI | -0.33 | 0.80 | -0.42 | 16.00 | 0.684 | Res. | 0.77 |
|  |  | Time | -0.31 | 0.26 | -1.19 | 17.00 | 0.250 |  |  |
|  | Foliar P | Int. | 1.20 | 2.12 | 0.57 | 17.12 | 0.578 | Plot | 8.63 |
|  |  | TPI | -3.77 | 2.91 | -1.30 | 16.00 | 0.213 | Res. | 2.34 |
|  |  | Time | 0.53 | 0.78 | 0.67 | 17.00 | 0.509 |  |  |
|  | Leaf area | Int. | 6.53 | 2.69 | 2.43 | 16.70 | 0.027 | Plot | 11.08 |
|  |  | TPI | 3.65 | 3.71 | 0.98 | 16.00 | 0.340 | Res. | 2.37 |
|  |  | Time | -0.29 | 0.79 | -0.37 | 17.00 | 0.714 |  |  |
|  | Leaf toughness | Int. | -2.42 | 0.30 | -8.07 | 28.53 | 0.000 | Plot | 0.76 |
|  |  | TPI | -0.68 | 0.35 | -1.97 | 16.00 | 0.067 | Res. | 1.01 |
|  |  | Time | -0.06 | 0.34 | -0.17 | 17.00 | 0.864 |  |  |
|  | Sapwood-specific conductivity [KS] | Int. | -0.78 | 1.34 | -0.58 | 18.86 | 0.567 | Plot | 5.16 |
|  |  | TPI | -3.11 | 1.79 | -1.74 | 16.00 | 0.101 | Res. | 2.31 |
|  |  | Time | -0.97 | 0.77 | -1.26 | 17.00 | 0.226 |  |  |
|  | Specific leaf area | Int. | -0.70 | 0.89 | -0.79 | 17.79 | 0.439 | Plot | 3.52 |
|  |  | TPI | 0.89 | 1.20 | 0.74 | 16.00 | 0.471 | Res. | 1.22 |
|  |  | Time | -0.04 | 0.41 | -0.09 | 17.00 | 0.927 |  |  |
|  | Vessel density | Int. | 49.28 | 28.76 | 1.71 | 21.29 | 0.101 | Plot | 101.96 |
|  |  | TPI | 7.84 | 37.09 | 0.21 | 16.00 | 0.835 | Res. | 65.59 |
|  |  | Time | 18.47 | 21.86 | 0.84 | 17.00 | 0.410 |  |  |
|  | Vessel diameter | Int. | -3.05 | 0.19 | -16.22 | 21.14 | 0.000 | Plot | 0.67 |
|  |  | TPI | -0.08 | 0.24 | -0.34 | 16.00 | 0.742 | Res. | 0.42 |
|  |  | Time | 0.05 | 0.14 | 0.37 | 17.00 | 0.717 |  |  |
|  | Wood density [WSG] | Int. | -1.97 | 0.46 | -4.26 | 25.31 | 0.000 | Plot | 1.40 |
|  |  | TPI | -1.18 | 0.56 | -2.11 | 16.00 | 0.051 | Res. | 1.36 |
|  |  | Time | -0.15 | 0.45 | -0.34 | 17.00 | 0.737 |  |  |
|  | Community Thermal Index (CTI) | Int. | -2.68 | 0.75 | -3.60 | 17.25 | 0.002 | Plot | 3.02 |
|  |  | TPI | 1.21 | 1.02 | 1.19 | 16.00 | 0.251 | Res. | 0.87 |
|  |  | Time | 0.15 | 0.29 | 0.53 | 17.00 | 0.606 |  |  |
|  | Community Precipitation Index (CPI) | Int. | -2.23 | 0.53 | -4.18 | 17.83 | 0.001 | Plot | 2.12 |
|  |  | TPI | -0.42 | 0.72 | -0.58 | 16.00 | 0.572 | Res. | 0.75 |
|  |  | Time | 0.13 | 0.25 | 0.54 | 17.00 | 0.594 |  |  |
