# Supplementary material for: Changes in tree functional composition across topographic gradients and through time in a tropical montane forest
Source: PLoS One. 2022 Apr 20;17(4):e0263508. doi: 10.1371/journal.pone.0263508 (PMC9020722; doi:10.1371/journal.pone.0263508)
Supplement: S5 Table — In the models, plot was included as a random effect. Data was collected in 18 permanent plots over eight years in Southern Ecuador. Statistically significant values are presented in bold. (DOCX) [file pone.0263508.s005.docx]

**S5 Table.** **Linear mixed models of community-weighted means (CWM) of ten functional traits and two climatic indices as a function of demographic grouping (i.e., recruited, dead, or growing trees), plot topographic position (Topographic Position Index= TPI) and their interaction.** In the models, plot was included as a random effect. Data was collected in 18 permanent plots over eight years in Southern Ecuador. Statistically significant values are presented in bold.

| **Functional trait /**  **Community climatic index** | **TPI** | | **Demography** | | **TPI x**  **Demography** | | **Random**  **effects** | | **Model** |
| --- | --- | --- | --- | --- | --- | --- | --- | --- | --- |
|  | F | P | F | P | F | P | Plot | Residual | R^2^ |
| Bark thickness | 4.80 | **0.044** | 0.40 | 0.675 | 0.56 | 0.577 | 0.00 | 0.12 | 0.09 |
| Foliar N | 69.00 | **<0.001** | 0.80 | 0.456 | 0.01 | 0.987 | 0.05 | 0.19 | 0.78 |
| Foliar P | 41.97 | **<0.001** | 0.92 | 0.409 | 1.00 | 0.379 | 0.05 | 0.29 | 0.67 |
| Leaf area [LA] | 8.41 | **0.011** | 0.90 | 0.416 | 1.60 | 0.217 | 0.04 | 0.07 | 0.62 |
| Leaf toughness | 35.40 | **<0.001** | 0.66 | 0.523 | 0.69 | 0.509 | 0.08 | 0.22 | 0.71 |
| Specific leaf area [SLA] | 56.96 | **<0.001** | 1.26 | 0.298 | 0.02 | 0.985 | 0.03 | 0.30 | 0.66 |
| Sapwood-specific conductivity (KS) | 6.82 | **0.019** | 0.68 | 0.512 | 2.10 | 0.139 | -0.02 | 0.50 | 0.14 |
| Vessel diameter | 8.62 | **0.010** | 4.17 | **0.025** | 0.19 | 0.832 | 0.00 | 0.23 | 0.28 |
| Vessel density | 2.94 | 0.106 | 1.20 | 0.314 | 1.33 | 0.278 | 0.01 | 0.22 | 0.18 |
| Wood density [WSG] | 48.79 | **<0.001** | 1.38 | 0.266 | 0.25 | 0.777 | 0.01 | 0.23 | 0.59 |
| Community temperature index (CTI) | 42.58 | **<0.001** | 3.14 | 0.057 | 0.56 | 0.574 | -0.16 | 1.94 | 0.36 |
| Community precipitation index (CPI) | 8.95 | **0.008** | 0.72 | 0.492 | 0.75 | 0.480 | -4406.92 | 37064.39 | 0.11 |
