# Supplementary material for: Changes in tree functional composition across topographic gradients and through time in a tropical montane forest
Source: PLoS One. 2022 Apr 20;17(4):e0263508. doi: 10.1371/journal.pone.0263508 (PMC9020722; doi:10.1371/journal.pone.0263508)
Supplement: S6 Table — (DOCX) [file pone.0263508.s006.docx]

**S6 Table.** **Methods used to assess functional traits..**

All selected trees to measure functional traits measured ≥ 10 cm DBH, were located within permanent plots or along the trail system of the reserve (1000 ha), and were collected between 2008 and 2014. Each trait was the measure of one individual or average of measures up to 10 individuals. Leaf traits were measured in five leaves from crown branches. We selected the youngest fully developed, sun-exposed, intact leaves. The “Function” field indicates the relation of the trait with plant physiology and performance according to selected studies conducted in tropical forests.

| **Trait** | **Unit** | **Function** | **Method** |
| --- | --- | --- | --- |
| Bark thickness (**BT**) | cm | Defense | Trunk bark thickness (cm) was measured with a bark gauge (Haglöf, Sweden) at 130 cm height. |
| Foliar nutrients (**N**, **P**) | mg g^-1^ | Photosynthetic capacity  (Kraft et al. 2010, Werner and Homeier 2015, Van der Sande et al. 2016) | Samples leaves were sent to the laboratory of the Department of Plant Ecology in Göttingen, Germany, and analyzed for foliar N concentrations using a C/N elemental analyzer (Vario EL III, Elementa, Germany) and for foliar P concentrations using Inductively Coupled Plasma Analysis (Optima 5300DV ICP-OES, Perkin Elmer, Waltham, Massachusetts, USA) after digesting the leaf samples with HNO_3_. |
| Leaf area (**LA**) | cm^2^ | Light interception, heat balance (Kraft et al. 2010, Van der Sande et al. 2016) | Fresh leaves were scanned with a flatbed scanner at 150 dpi and average leaf area was calculated with WinFOLIA 2005b software (Regent Instruments, Inc., Canada). |
| Leaf toughness (**LT**) | kN m^-1^ | Defense | Leaf toughness was estimated as the mean of six punch tests using a digital penetrometer (2.0 mm diameter, DS-20N, Imada Inc., Japan) on three fresh leaves (excluding the midrib and other major veins) from each tree (following Onoda et al. 2011, Katabuchi et al. 2012). |
| Specific leaf area (**SLA**) | cm^2^ g^-1^ | Resource capture  (Kraft et al. 2010, Van der Sande et al. 2016) | The scanned leaves were then dried at 60°C for 72 hours and the dry weight was used to calculate the specific leaf area. |
| Sapwood-specific conductivity (**KS**) | kg m^-1^ MPa^-1^ s^-1^ | Conductivity of fluids, drought tolerance  (Sedio et al. 2012) | Theoretical sapwood-specific conductivity was calculated according to the Hagen-Poiseuille equation (following Kotowska et al. 2015). |
| Vessel density (**VDens**) | mm^-2^ | Conductivity of fluids, drought tolerance  (Sedio et al. 2012) | Dried samples were then stored in ethanol (70%) and sent to the laboratory of the Department of Plant Ecology in Göttingen (Germany), where the material was analyzed for additional wood traits. The wood cores were stained with Safranin solution. Cross sections were cut with a sliding microtome (G.S.L.1, WSL, Switzerland). Photographs of the cross-sectional cuts were taken with a stereo-microscope with digital camera (Zeiss SteREOV20, Gemany). Image processing was done with Adobe Photoshop CS2 (Adobe Systems Incorporated, USA) and ImageJ software (https://imagej.nih.gov/ij/) using the particle analysis-function for estimating mean vessel density. |
| Vessel diameter (**VDia**) | µm | Conductivity of fluids, drought tolerance  (Sedio et al. 2012) | Dried samples were then stored in ethanol (70%) and sent to the laboratory of the Department of Plant Ecology in Göttingen (Germany), where the material was analyzed for additional wood traits. The wood cores were stained with Safranin solution. Cross sections were cut with a sliding microtome (G.S.L.1, WSL, Switzerland). Photographs of the cross-sectional cuts were taken with a stereo-microscope with digital camera (Zeiss SteREOV20, Gemany). Image processing was done with Adobe Photoshop CS2 (Adobe Systems Incorporated, USA) and ImageJ software (https://imagej.nih.gov/ij/) using the particle analysis-function for estimating mean vessel diameter. |
| Wood specific gravity (**WSG**) | No unit | Mechanical strength, stem defense, drought tolerance  (Valencia et al. 2009, Kraft et al. 2010, Van der Sande et al. 2016) | Wood cores (5 mm diameter, 5 cm length) of the outer wood were taken at 1 m above ground with an increment corer (Suunto, Finland), volume of the fresh samples was determined and samples were subsequently dried at 102°C for 72 hours to determine wood dry mass. Wood specific gravity (WSG) was calculated as the sample dry mass divided by sample fresh volume. |

**References**

Katabuchi, M., Kurokawa, H., Davies, S.J., Tan, S., & Nakashizuka, T. (2012). Soil resource availability shapes community trait structure in a species‐rich dipterocarp forest. Journal of Ecology, 100(3), 643-651.

Kotowska, M. M., Hertel, D., Abou Rajab, Y., Barus, H. & Schuldt, B. (2015). Patterns in hydraulic architecture from roots to branches in six tropical tree species from cacao agroforestry and their relation to wood density and stem growth. Front. Plant Sci. 6:191.

Onoda, Y., Westoby, M., Adler, P. B., Choong, A. M., Clissold, F. J., Cornelissen, J. H. et al. (2011) Global patterns of leaf mechanical properties. Ecology Letters, 14(3), 301-312.

Sedio, B. E., Wright, S. J. & Dick, C. W. (2012) Trait evolution and the coexistence of a species swarm in the tropical forest understorey. *Journal of Ecology,* **100,** 1183-1193.

Valencia, R., Condit, R., Muller-Landau, H. C., Hernandez, C. & Navarrete, H. (2009) Dissecting biomass dynamics in a large Amazonian forest plot. *Journal of Tropical Ecology,* **25,** 473-482.

van der Sande, M. T., Arets, E. J. M. M., Peña-Claros, M., de Avila, A. L., Roopsind, A., Mazzei, L., Ascarrunz, N., Finegan, B., Alarcón, A., Cáceres-Siani, Y., Licona, J. C., Ruschel, A., Toledo, M. & Poorter, L. (2016) Old-growth Neotropical forests are shifting in species and trait composition. *Ecological Monographs,* **86,** 228–243.

Werner, F. A. & Homeier, J. (2015) Is tropical montane forest heterogeneity promoted by a resource-driven feedback cycle? Evidence from nutrient relations, herbivory and litter decomposition along a topographical gradient. *Functional Ecology,* **29,** 430–440.
