# Supplementary material for: Changes in tree functional composition across topographic gradients and through time in a tropical montane forest
Source: PLoS One. 2022 Apr 20;17(4):e0263508. doi: 10.1371/journal.pone.0263508 (PMC9020722; doi:10.1371/journal.pone.0263508)
Supplement: S7 Table — Figures are calculated for 18 20 x 20 m forest plots in Southern Ecuador. (DOCX) [file pone.0263508.s007.docx]

**S7 Table.** **Mean and standard error of the number of species with trait values, and percentage of plot scale basal area with trait values for the first and last year of our monitoring period.**

|  | **Year 1** | | **Year 8** | |
| --- | --- | --- | --- | --- |
| Trait/Community climatic index | Number of species with trait data (SE) | % basal area (SE) | Number of species with trait data (SE) | % basal area (SE) |
| Bark thickness [BT] | 26.55 (0.95) | 83.26 (2.27) | 24.88 (1.34) | 83.91 (1.98) |
| Leaf area [LA] | 26.5 (1.0) | 81.03 (1.83) | 24.94 (1.42) | 82.07 (1.84) |
| Leaf toughness [LT] | 26.5 (1.0) | 81.03 (1.83) | 24.94 (1.42) | 82.07 (1.84) |
| Foliar nitrogen [N] | 21.94 (0.95) | 59.52 (4.64) | 20.77 (1.33) | 60.28 (5.06) |
| Foliar phosphorus [P] | 22.88 (1.01) | 69.69 (3.29) | 21.61 (1.41) | 70.66 (3.60) |
| Sapwood-specific conductivity [KS] | 28.05 (1.07) | 85.65 (1.83) | 26.27 (1.57) | 86.16 (1.65) |
| Specific leaf area [SLA] | 24.22 (0.97) | 73.23 (3.21) | 22.77 (1.39) | 73.81 (3.38) |
| Vessel density [VDen] | 28.05 (1.07) | 85.65 (1.83) | 26.27 (1.57) | 86.16 (1.65) |
| Vessel diameter[VDia] | 28.05 (1.07) | 85.65 (1.83) | 26.27 (1.57) | 86.16 (1.65) |
| Wood density [WSG] | 28.44 (1.06) | 85.93 (1.76) | 26.77 (1.54) | 86.44 (1.59) |
| Community temperature index [CTI] | 20.5 (0.94) | 70.36 (2.52) | 19.33 (1.25) | 70.02 (2.40) |
| Community precipitation index [CPI] | 20.5 (0.94) | 70.36 (2.52) | 19.33 (1.25) | 70.02 (2.40) |
