# Supplementary material for: Changes in tree functional composition across topographic gradients and through time in a tropical montane forest
Source: PLoS One. 2022 Apr 20;17(4):e0263508. doi: 10.1371/journal.pone.0263508 (PMC9020722; doi:10.1371/journal.pone.0263508)
Supplement: S8 Table — (DOCX) [file pone.0263508.s008.docx]

**S8 Table. Results of correlation analyses comparing community-weighted mean values calculated using plot scale tree basal area to plot-scale number of individual stems for trees at the beginning and the end of the monitoring period (year 1 and year 8, respectively), and for each tree demographic group.**

| **Time/demographic group** | **Trait** | **Correlation coefficient** | **P** |
| --- | --- | --- | --- |
| Year 1 | Bark thickness [BT] | 0.40 | 0.160 |
|  | Leaf area [LA] | 0.91 | 0.000 |
|  | Leaf toughness [LT] | 0.96 | 0.000 |
|  | Foliar nitrogen [N] | 0.99 | 0.000 |
|  | Foliar phosphorus [P] | 0.99 | 0.000 |
|  | Sapwood-specific conductivity [KS] | 0.31 | 0.277 |
|  | Specific leaf area [SLA] | 0.98 | 0.000 |
|  | Vessel density [VDen] | -0.37 | 0.193 |
|  | Vessel diameter[VDia] | 0.83 | 0.000 |
|  | Wood density [WSG] | 0.94 | 0.000 |
|  | Community thermal index [CTI] | 0.97 | 0.000 |
|  | Community precipitation index [CPI] | 0.86 | 0.000 |
|  | **mean** | **0.73** | **0.053** |
| Year 8 | Bark thickness [BT] | 0.61 | 0.021 |
|  | Leaf area [LA] | 0.86 | 0.000 |
|  | Leaf toughness [LT] | 0.95 | 0.000 |
|  | Foliar nitrogen [N] | 0.99 | 0.000 |
|  | Foliar phosphorus [P] | 0.99 | 0.000 |
|  | Sapwood-specific conductivity [KS] | 0.61 | 0.021 |
|  | Specific leaf area [SLA] | 0.99 | 0.000 |
|  | Vessel density [VDen] | 0.29 | 0.315 |
|  | Vessel diameter[VDia] | 0.74 | 0.003 |
|  | Wood density [WSG] | 0.98 | 0.000 |
|  | Community thermal index [CTI] | 0.98 | 0.000 |
|  | Community precipitation index [CPI] | 0.93 | 0.000 |
|  | **mean** | **0.83** | **0.030** |
| Growth | Bark thickness [BT] | 0.88 | 0.000 |
|  | Leaf area [LA] | 0.91 | 0.000 |
|  | Leaf toughness [LT] | 0.98 | 0.000 |
|  | Foliar nitrogen [N] | 1.00 | 0.000 |
|  | Foliar phosphorus [P] | 1.00 | 0.000 |
|  | Sapwood-specific conductivity [KS] | 0.59 | 0.026 |
|  | Specific leaf area [SLA] | 1.00 | 0.000 |
|  | Vessel density [VDen] | 0.51 | 0.063 |
|  | Vessel diameter[VDia] | 0.91 | 0.000 |
|  | Wood density [WSG] | 0.97 | 0.000 |
|  | Community thermal index [CTI] | 0.96 | 0.000 |
|  | Community precipitation index [CPI] | 0.91 | 0.000 |
|  | **mean** | **0.89** | **0.007** |
| Mortality | Bark thickness [BT] | 0.66 | 0.010 |
|  | Leaf area [LA] | 0.98 | 0.000 |
|  | Leaf toughness [LT] | 0.99 | 0.000 |
|  | Foliar nitrogen [N] | 0.99 | 0.000 |
|  | Foliar phosphorus [P] | 0.97 | 0.000 |
|  | Sapwood-specific conductivity [KS] | 0.83 | 0.000 |
|  | Specific leaf area [SLA] | 0.99 | 0.000 |
|  | Vessel density [VDen] | 0.92 | 0.000 |
|  | Vessel diameter[VDia] | 0.73 | 0.003 |
|  | Wood density [WSG] | 0.90 | 0.000 |
|  | Community thermal index [CTI] | 0.97 | 0.000 |
|  | Community precipitation index [CPI] | 0.97 | 0.000 |
|  | **mean** | **0.91** | **0.001** |
| Recruitment | Bark thickness [BT] | 0.99 | 0.000 |
|  | Leaf area [LA] | 0.99 | 0.000 |
|  | Leaf toughness [LT] | 0.99 | 0.000 |
|  | Foliar nitrogen [N] | 1.00 | 0.000 |
|  | Foliar phosphorus [P] | 1.00 | 0.000 |
|  | Sapwood-specific conductivity [KS] | 1.00 | 0.000 |
|  | Specific leaf area [SLA] | 1.00 | 0.000 |
|  | Vessel density [VDen] | 0.99 | 0.000 |
|  | Vessel diameter[VDia] | 0.99 | 0.000 |
|  | Wood density [WSG] | 1.00 | 0.000 |
|  | Community thermal index [CTI] | 0.99 | 0.000 |
|  | Community precipitation index [CPI] | 0.96 | 0.000 |
|  | **mean** | **0.99** | **0.000** |
