# Supplementary material for: Changes in tree functional composition across topographic gradients and through time in a tropical montane forest
Source: PLoS One. 2022 Apr 20;17(4):e0263508. doi: 10.1371/journal.pone.0263508 (PMC9020722; doi:10.1371/journal.pone.0263508)
Supplement: S9 Table — (DOCX) [file pone.0263508.s009.docx]

**S9 Table. Methods used to evaluate the relevance of elevation and topography (TPI) on community floristic composition.**

We conducted PERMANOVAs predicting plot level species composition as a function of Topographic Position Index (TPI) or elevation, and compared the R^2^ and the *P* values of the models. For each pair of models, highest R^2^ and lowest *P* values (when applicable) are in bold. These analyses showed that TPI was a stronger predictor than elevation as the resulting models consistently had higher R^2^ and lower *P* values.

Table 1) Models testing for the effects of topography or elevation on species composition during the first year of the monitoring period.

| Factor | d.f. | SS | MS | *F* | R^2^ | *P* |
| --- | --- | --- | --- | --- | --- | --- |
| TPI | 1 | 1.53 | 1.53 | 4.54 | **0.221** | **0.001** |
| Residuals | 16 | 5.38 | 0.34 | 0.78 |  |  |
| Total | 17 | 6.91 | 1.00 |  |  |  |
| Elevation | 1 | 1.22 | 1.22 | 3.45 | 0.177 | **0.001** |
| Residuals | 16 | 5.68 | 0.36 | 0.82 |  |  |
| Total | 17 | 6.91 | 1.00 |  |  |  |

Table 2) Models testing for the effects of topography or elevation on species composition over time.

| Factor | d.f. | SS | MS | *F* | R^2^ | *P* |
| --- | --- | --- | --- | --- | --- | --- |
| Time | 1 | 0.02 | 0.02 | 0.06 | 0.002 | 1.000 |
| TPI | 1 | 3.07 | 3.07 | 9.15 | **0.222** | **0.001** |
| Time x TPI | 1 | 0.02 | 0.02 | 0.07 | 0.002 | 1.000 |
| Residuals | 32 | 10.72 | 0.34 | 0.78 |  |  |
| Total | 35 | 13.84 | 1.00 |  |  |  |
| Time | 1 | 0.02 | 0.02 | 0.06 | 0.002 | 1.000 |
| Elevation | 1 | 2.42 | 2.42 | 6.82 | 0.175 | **0.001** |
| Time x elevation | 1 | 0.03 | 0.03 | 0.08 | 0.002 | 1.000 |
| Residuals | 32 | 11.37 | 0.36 | 0.82 |  |  |
| Total | 35 | 13.84 | 1.00 |  |  |  |

Table 3) Models testing for the effects of topography or elevation on species composition across demographic groups (i.e., dead, surviving, and recruited trees).

| Factor | d.f. | SS | MS | *F* | R^2^ | *P* |
| --- | --- | --- | --- | --- | --- | --- |
| Status | 1 | 0.71 | 0.71 | 1.68 | **0.044** | **0.004** |
| TPI | 1 | 1.30 | 1.30 | 3.07 | **0.080** | **0.001** |
| Demographic group x topography | 1 | 0.58 | 0.58 | 1.37 | **0.036** | **0.028** |
| Residuals | 32 | 13.60 | 0.43 | 0.84 |  |  |
| Total | 35 | 16.20 | 1.00 |  |  |  |
| Status | 1 | 0.71 | 0.71 | 1.56 | 0.044 | 0.004 |
| Elevation | 1 | 0.47 | 0.47 | 1.02 | 0.029 | 0.430 |
| Demographic group x elevation | 1 | 0.44 | 0.44 | 0.96 | 0.027 | 0.586 |
| Residuals | 32 | 14.59 | 0.46 | 0.90 |  |  |
| Total | 35 | 16.20 | 1.00 |  |  |  |
