# Supplementary material for: Changes in tree functional composition across topographic gradients and through time in a tropical montane forest
Source: PLoS One. 2022 Apr 20;17(4):e0263508. doi: 10.1371/journal.pone.0263508 (PMC9020722; doi:10.1371/journal.pone.0263508)
Supplement: S10 Table — (DOCX) [file pone.0263508.s010.docx]

**S10 Table. Methods used to evaluate the relevance of elevation and topography (TPI) on community climate indices (CTI and CPI) and community weighted means.**

We used Akaike Information Criterion (AIC) to compare linear mixed models that predicted community trait composition for each trait (CWMs) and community climate indices (CTI and CPI) as a function of TPI, time, TPI x time, and elevation (with plot as a random effect), with models that did not include elevation. Our results showed that for all our functional traits and for CTI, models without elevation had lower AIC values (Δ AIC >2) or were equally informative (ΔAIC <2) than those that included it (Table S1), indicating that elevation did not provide substantial additional information. This result did not hold for CPI, as the best model did include elevation. However, further examination of the data showed that the validity of the models decreased (ΔAIC values increased by >2) when elevation replaced TPI in the model (elevation x year), or if TPI was included as a model covariate (elevation x year + TPI). Furthermore, linear regressions showed that TPI had statistically significant effects on the community composition of all functional traits and community climatic indices (Table S2), whereas elevation affected seven functional traits and one community climatic index (Table S3).

Table 1) Akaike Information Criterion (AIC) of Linear Mixed Models predicting community trait composition (calculated as community weighted means) and Community climatic indices as a function of Topographic Position Index (TPI) and time, with and without the elevation factor as a covariate. Plot was included as a random factor in both types of models. For each functional trait and community climatic index, the lowest AIC values are in bold.

| Trait / Community climatic index | AIC  TPI x time + elevation (*df* 7) | AIC  TPI x time (*df* 6) | Δ AIC |
| --- | --- | --- | --- |
| Bark thickness [BT] | 17.89 | **12.83** | 5.05 |
| Leaf area [LA] | 14.16 | **11.56** | 2.60 |
| Leaf toughness [LT] | 48.49 | **46.07** | 2.42 |
| Foliar nitrogen [N] | 24.72 | **20.96** | 3.76 |
| Foliar phosphorus [P] | 28.89 | **28.21** | 0.68 |
| Sapwood-specific conductivity [KS] | 45.71 | **41.35** | 4.35 |
| Specific leaf area [SLA] | 34.13 | **33.06** | 1.07 |
| Vessel density [VDen] | -2.70 | **-9.05** | 6.35 |
| Vessel diameter[VDia] | 38.12 | **34.42** | 3.71 |
| Wood density [WSG] | 15.09 | **14.56** | 0.53 |
| Community thermal index (CTI) | 96.23 | **93.88** | 2.35 |
| Community precipitation index (CPI) | **390.37** | 399.76 | 9.38 |

Table 2) Linear regressions of Community weighted means for 18 plots, during the first and last year of the study, as a function of topography (measured as Topographic Position Index, TPI). Statistical significant values are in bold.

| Functional trait/  Community climatic Index | **Year 1** | | | | **Year 8** | | | |
| --- | --- | --- | --- | --- | --- | --- | --- | --- |
|  | Estimate | SE | *P* | R^2^ | Estimate | SE | *P* | R^2^ |
| Bark thickness [BT] | -0.18 | 0.08 | **0.047** | 0.23 | -0.23 | 0.08 | **0.014** | 0.32 |
| Leaf area [LA] | -0.82 | 0.10 | **<0.001** | 0.81 | -0.87 | 0.09 | **<0.001** | 0.84 |
| Leaf toughness [LT] | -0.79 | 0.13 | **<0.001** | 0.71 | -0.81 | 0.15 | **<0.001** | 0.65 |
| Foliar nitrogen [N] | -0.35 | 0.13 | **0.016** | 0.31 | -0.29 | 0.13 | **0.044** | 0.23 |
| Foliar phosphorus [P] | 0.49 | 0.16 | **0.007** | 0.79 | 0.77 | 0.12 | **<0.001** | 0.73 |
| Sapwood-specific conductivity [KS] | -0.82 | 0.12 | **0.000** | 0.75 | -0.88 | 0.13 | **<0.001** | 0.74 |
| Specific leaf area [SLA] | -0.23 | 0.11 | **0.042** | 0.23 | -0.32 | 0.12 | **0.020** | 0.29 |
| Vessel density [VDen] | -0.34 | 0.13 | **0.021** | 0.29 | -0.33 | 0.13 | **0.027** | 0.27 |
| Vessel diameter[VDia] | 0.15 | 0.05 | **0.012** | 0.33 | 0.10 | 0.04 | **0.019** | 0.3 |
| Wood density [WSG] | 0.61 | 0.10 | **<0.001** | 0.68 | 0.64 | 0.09 | **<0.001** | 0.76 |
| Community temperature  index (CTI) | -1.60 | 0.30 | **<0.001** | 0.64 | -1.39 | 0.32 | **0.001** | 0.54 |
| Community precipitation  index (CPI) | -138.61 | 50.43 | **0.014** | 0.32 | -113.09 | 52.01 | **0.045** | 0.23 |

Table 3) Linear regressions of Community weighted means for 18 plots, during the first and last year of the study, as a function of elevation. Statistical significant values are in bold.

| Functional trait /  Community climatic index | **Year 1** | | | | **Year 8** | | | |
| --- | --- | --- | --- | --- | --- | --- | --- | --- |
|  | Estimate | SE | *P* | R^2^ | Estimate | SE | *P* | R^2^ |
| Bark thickness [BT] | 0.00 | 0.00 | 0.106 | 0.16 | 0.00 | 0.00 | 0.141 | 0.13 |
| Leaf area [LA] | -0.01 | 0.00 | **<0.001** | 0.60 | -0.01 | 0.00 | **<0.001** | 0.56 |
| Leaf toughness [LT] | -0.01 | 0.00 | **<0.001** | 0.57 | -0.01 | 0.00 | **0.002** | 0.46 |
| Foliar nitrogen [N] | 0.00 | 0.00 | **0.043** | 0.23 | 0.00 | 0.00 | 0.063 | 0.20 |
| Foliar phosphorus [P] | 0.01 | 0.00 | **<0.001** | 0.66 | 0.01 | 0.00 | **<0.001** | 0.55 |
| Sapwood-specific conductivity [KS] | -0.01 | 0.00 | **<0.001** | 0.65 | -0.01 | 0.00 | **<0.001** | 0.55 |
| Specific leaf area [SLA] | 0.00 | 0.00 | 0.216 | 0.09 | 0.00 | 0.00 | **0.026** | 0.27 |
| Vessel density [VDen] | 0.00 | 0.00 | 0.051 | 0.22 | 0.00 | 0.00 | **0.042** | 0.23 |
| Vessel diameter[VDia] | 0.00 | 0.00 | **0.031** | 0.26 | 0.00 | 0.00 | 0.468 | 0.03 |
| Wood density [WSG] | 0.01 | 0.00 | **<0.001** | 0.61 | 0.01 | 0.00 | **<0.001** | 0.64 |
| Community temperature  index (CTI) | -0.02 | 0.01 | **0.011** | 0.34 | -0.02 | 0.01 | **0.013** | 0.33 |
| Community precipitation  index (CPI) | -0.78 | 0.86 | 0.376 | 0.08 | -0.58 | 0.84 | 0.502 | 0.03 |
