# Supplementary material for: Changes in tree functional composition across topographic gradients and through time in a tropical montane forest
Source: PLoS One. 2022 Apr 20;17(4):e0263508. doi: 10.1371/journal.pone.0263508 (PMC9020722; doi:10.1371/journal.pone.0263508)
Supplement: S11 Table — (DOCX) [file pone.0263508.s011.docx]

**S11 Table. Methods and results of analyses testing for spatial autocorrelation in trait composition and community climatic indices of 18 plots in southern Ecuador**

Methods: We used Akaike Information Criteria to compare simple linear models predicting trait composition as a function of Topograhic Position Index (TPI) to equivalent models that incorporated three types of spatial signals: Spherical Correlation, Gaussian, and Exponential following the modelling techniques in Zuur et al. 2009 (pp. 161-170). Results: none of the traits the models including a spatial autocorrelation component were consistently better (had ΔAIC value > 6) than simple linear models. We therefore did not find evidence that including spatial autocorrelation in further statistical models was needed.

| **Trait/Community climatic index** | **Model** | **df** | **AIC** | Δ **AIC** |
| --- | --- | --- | --- | --- |
| Bark thickness | **No spatial** | **3** | **12.6** | **0.0** |
|  | Spherical correlation structure | 5 | 16.6 | 4.0 |
|  | Gaussian | 5 | 16.6 | 4.0 |
|  | Explonential | 5 | 16.6 | 4.0 |
| Foliar N | **No spatial** | **3** | **18.3** | **0.0** |
|  | Spherical correlation structure | 5 | 22.3 | 4.0 |
|  | Gaussian | 5 | 21.2 | 2.9 |
|  | Explonential | 5 | 21.7 | 3.4 |
| Foliar P | **No spatial** | **3** | **26.0** | **0.0** |
|  | Spherical correlation structure | 5 | 30.0 | 4.0 |
|  | Gaussian | 5 | 30.0 | 4.0 |
|  | Explonential | 5 | 30.0 | 4.0 |
| Leaf area | **No spatial** | **3** | **26.2** | **0.0** |
|  | Spherical correlation structure | 5 | 30.2 | 4.0 |
|  | Gaussian | 5 | 30.2 | 4.0 |
|  | Explonential | 5 | 30.2 | 4.0 |
| Leaf toughness | **No spatial** | **3** | **25.0** | **0.0** |
|  | Spherical correlation structure | 5 | 29.0 | 4.0 |
|  | Gaussian | 5 | 29.0 | 4.0 |
|  | Explonential | 5 | 29.0 | 4.0 |
| Sapwood-specific conductivity | No spatial | 3 | 20.0 | 3.8 |
|  | Spherical correlation structure | 5 | 18.4 | 2.2 |
|  | **Gaussian** | 5 | **16.2** | **0.0** |
|  | Explonential | 5 | 20.3 | 4.1 |
| Specific leaf area | **No spatial** | **3** | **23.5** | **0.0** |
|  | Spherical correlation structure | 5 | 27.4 | 3.9 |
|  | Gaussian | 5 | 27.5 | 4.0 |
|  | Explonential | 5 | 27.3 | 3.8 |
| Vessel density | **No spatial** | **3** | **27.0** | **0.0** |
|  | Spherical correlation structure | 5 | 28.9 | 2.0 |
|  | Gaussian | 5 | 28.6 | 1.6 |
|  | Explonential | 5 | 29.2 | 2.2 |
| Vessel diameter | **No spatial** | **3** | **-1.8** | **0.0** |
|  | Spherical correlation structure | 5 | 2.2 | 4.0 |
|  | Gaussian | 5 | 2.2 | 4.0 |
|  | Explonential | 5 | 2.2 | 4.0 |
| Wood density | **No spatial** | **3** | **19.3** | **0.0** |
|  | Spherical correlation structure | 5 | 23.2 | 3.9 |
|  | Gaussian | 5 | 23.3 | 4.0 |
|  | Explonential | 5 | 21.6 | 2.3 |
| Community temperature index | **No spatial** | **3** | **53.7** | **0.0** |
|  | Spherical correlation structure | 5 | 57.7 | 4.0 |
|  | Gaussian | 5 | 56.0 | 2.3 |
|  | Explonential | 5 | 57.1 | 3.4 |
| Community precipitation index | No spatial | 3 | 217.3 | 2.5 |
|  | Spherical correlation structure | 5 | **214.9** | **0.0** |
|  | Gaussian | 5 | 215.1 | 0.2 |
|  | Explonential | 5 | 215.1 | 0.2 |

**Reference**

Zuur, A., Ieno, E., Walker, N., Saveliev, A. & Smith, G. (2009) *Mixed Effects Models and Extensions in Ecology with R*. Springer Sicence+ Business Media, LLC.
