# Supplementary material for: Changes in tree functional composition across topographic gradients and through time in a tropical montane forest
Source: PLoS One. 2022 Apr 20;17(4):e0263508. doi: 10.1371/journal.pone.0263508 (PMC9020722; doi:10.1371/journal.pone.0263508)

**S1 Fig. Non-metric multidimensional scaling of tree species composition during the first year of the study, in 18 permanent plots in southern Ecuador.** Topographic variation is expressed as a Topographic Position Index (TPI).


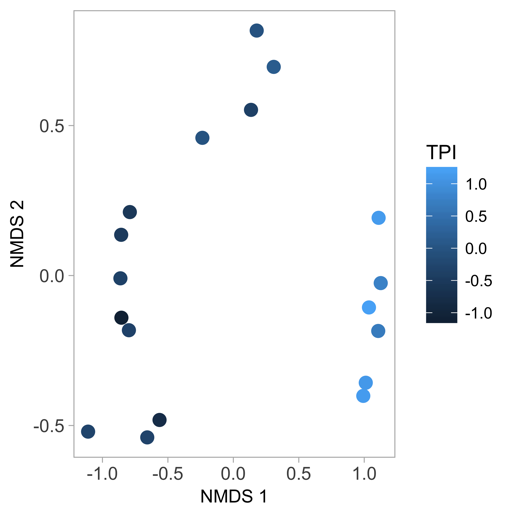

Supplement: S1 Fig — Topographic variation is expressed as a Topographic Position Index (TPI). (DOCX) [file pone.0263508.s012.docx]
