# Supplementary material for: Changes in tree functional composition across topographic gradients and through time in a tropical montane forest
Source: PLoS One. 2022 Apr 20;17(4):e0263508. doi: 10.1371/journal.pone.0263508 (PMC9020722; doi:10.1371/journal.pone.0263508)
Supplement: S2 Fig — Statistically significant relations (p<0.05) with Topographic Position Index (TPI) are indicated by solid regression lines. (DOCX) [file pone.0263508.s013.docx]

**S2 Fig. Linear mixed models testing for the effects of topography and time on community weighted moments (CWV, CWS, CWK) in eighteen permanent plots in Southern Ecuador.** Statistically significant relations (p<0.05) with Topographic Position Index (TPI) are indicated by solid regression lines.

**
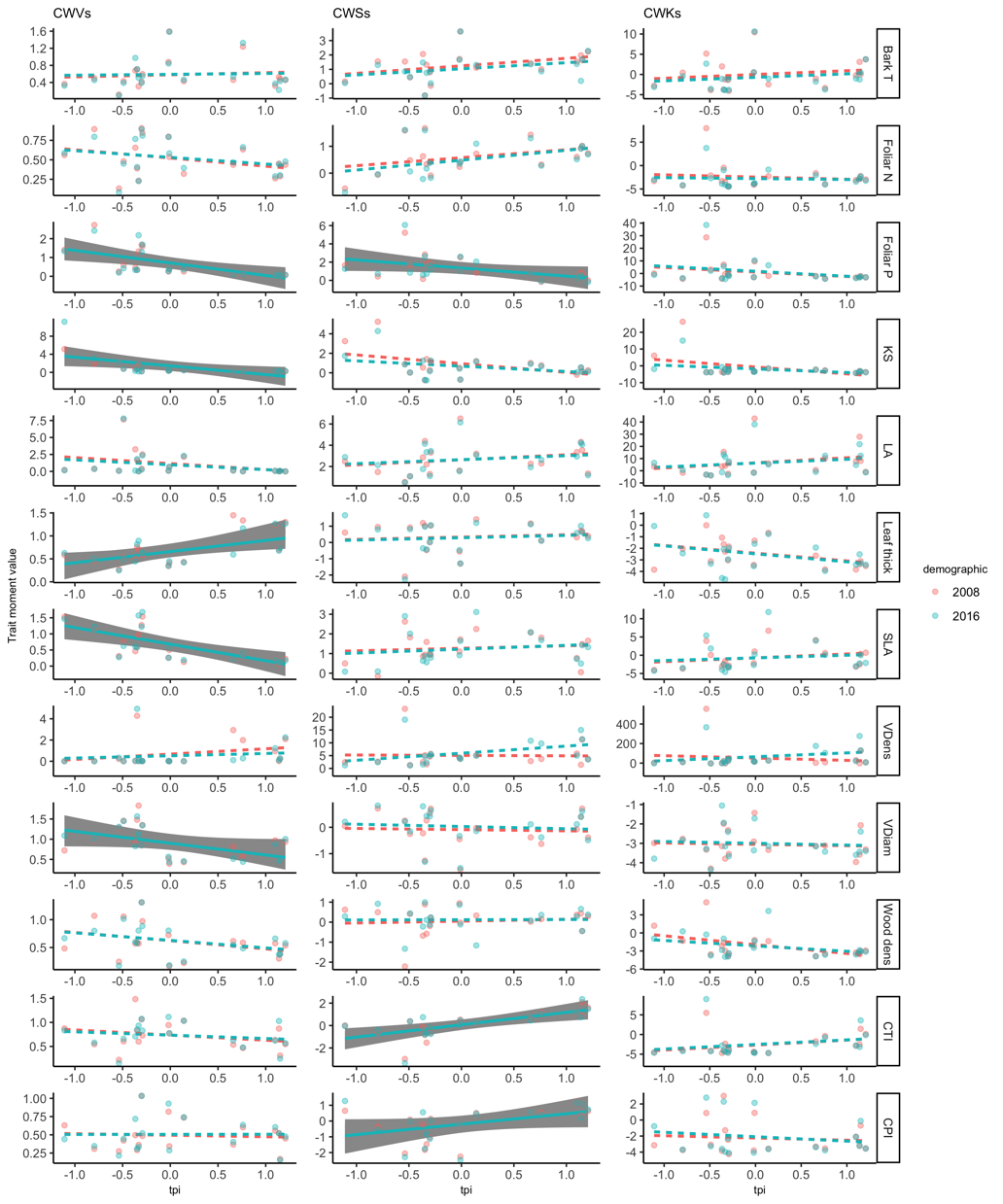
**
