# Supplementary material for: Changes in tree functional composition across topographic gradients and through time in a tropical montane forest
Source: PLoS One. 2022 Apr 20;17(4):e0263508. doi: 10.1371/journal.pone.0263508 (PMC9020722; doi:10.1371/journal.pone.0263508)

**S3 Fig.** **Functional trait composition as normalized Community Weighted Means (CWM) considering three demographic groups in 18 permanent plots over eight years; CWM are a function of Topographic Position Index (TPI).** Stars indicate statistically significant values at *P*≤0.05.


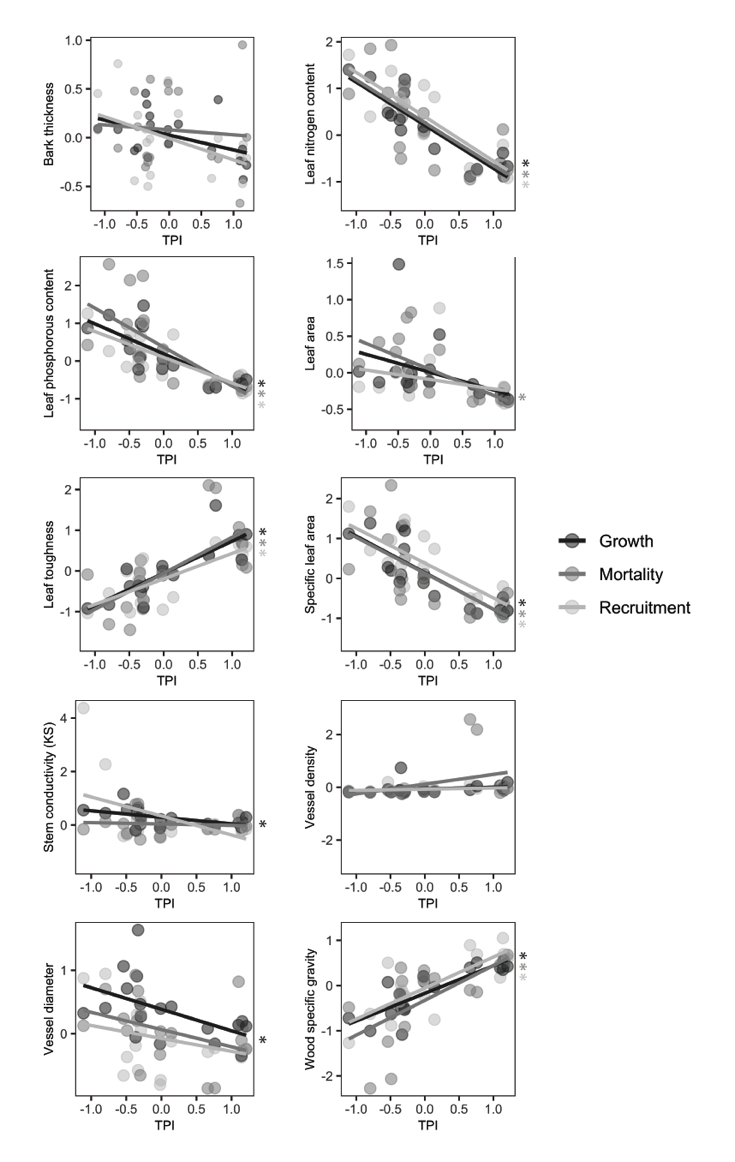

Supplement: S3 Fig — Stars indicate statistically significant values at P≤0.05. (DOCX) [file pone.0263508.s014.docx]
