# Supplementary material for: Changes in tree functional composition across topographic gradients and through time in a tropical montane forest
Source: PLoS One. 2022 Apr 20;17(4):e0263508. doi: 10.1371/journal.pone.0263508 (PMC9020722; doi:10.1371/journal.pone.0263508)
Supplement: S4 Fig — (DOCX) [file pone.0263508.s015.docx]

**S4 Fig.** **A) Image of the study area showing the rugged topography, B) the distribution of the 18 permanent study plots in the San Francisco reserve and C) schematic sketch showing the slope positions and their respective Topographic Position Index values (TPI), from most negative values at valley bottoms to most positive values at ridge tops.**

| A) |
| --- |
| 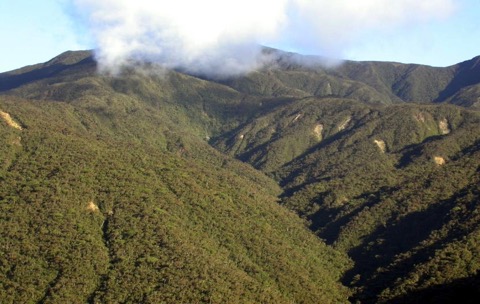 |
| B) |
| 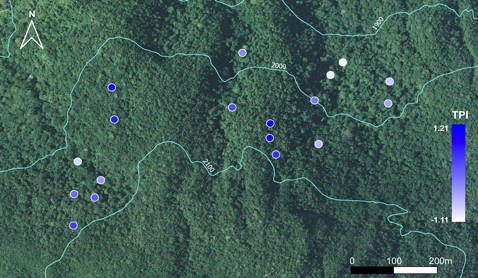 |
| C) |
| 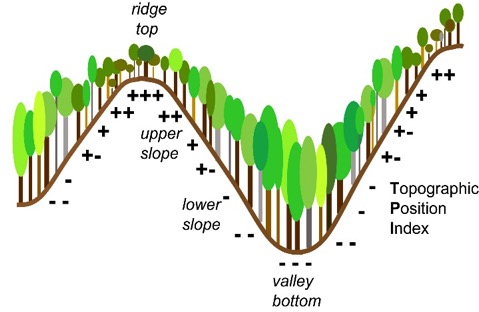 |
