# Supplementary material for: Changes in tree functional composition across topographic gradients and through time in a tropical montane forest
Source: PLoS One. 2022 Apr 20;17(4):e0263508. doi: 10.1371/journal.pone.0263508 (PMC9020722; doi:10.1371/journal.pone.0263508)
Supplement: S5 Fig — Soil properties: Depth of organic layer (dol), pH value, concentrations of K, Mg, Ca, Al and N, C:N ratio, plant available phosphorus (Pav), nitrogen mineralization rate (Nmin) and nitrogen nitrification rate (Nnitr). Data from Wolf et al. (2011) as presented in Pierick et al.(2021). All correlations are statistically significant, and shadows indicate 95% confidence intervals. (DOCX) [file pone.0263508.s016.docx]

**S5 Fig.** **Correlations of soil features with Topographic Position Index (TPI) in the 18 study plots.** Soil properties: Depth of organic layer (dol), pH value, concentrations of K, Mg, Ca, Al and N, C:N ratio, plant available phosphorus (Pav), nitrogen mineralization rate (Nmin) and nitrogen nitrification rate (Nnitr). Data from Wolf *et al. (*2011) as presented in Pierick et al.(2021). All correlations are statistically significant, and shadows indicate 95% confidence intervals.

**
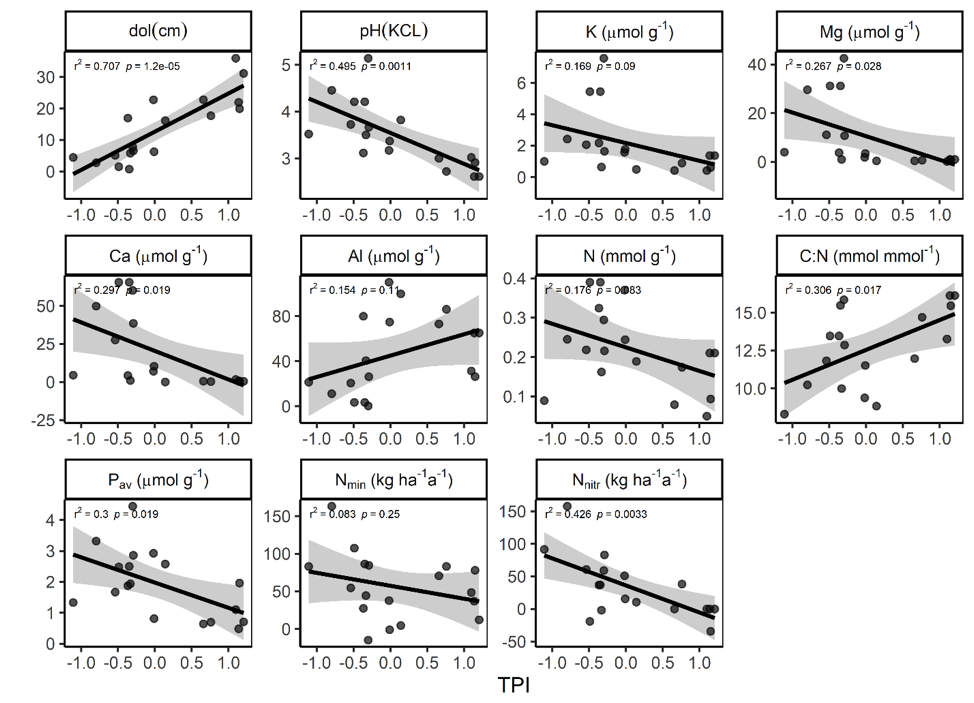
**

**Reference**

Wolf, K., et al. (2011). Nitrogen availability links forest productivity, soil nitrous oxide and nitric oxide fluxes of a tropical montane forest in southern Ecuador. Global Biogeochemical Cycles **25**(4): GB4009.

Pierick, K., et al. (2021). Topography as a factor driving small-scale variation in tree fine root traits and root functional diversity in a species-rich tropical montane forest. New Phytologist **230**(1): 129-138.
